# Oral contraceptives exposure may reduce the risk of ovarian cancer: a meta-analysis based on cohort studies

**DOI:** 10.3389/fphar.2026.1732719

**Published:** 2026-04-01

**Authors:** Qian Sun, Jiani Ji, Jingqi Chen, Ting Jiang, Shiyan Zhang, Hongli Yang

**Affiliations:** 1 Department of Gynecology, Ningbo Hospital of Integrated Traditional Chinese and Western Medicine, Ningbo, Zhejiang, China; 2 The Second Clinical Medical College, Zhejiang Chinese Medical University, Hangzhou, Zhejiang, China; 3 The First Affiliated Hospital of Zhejiang Chinese Medical University (Zhejiang Provincial Hospital of Chinese Medicine), Hangzhou, Zhejiang, China

**Keywords:** incidence, meta-analysis, oral contraceptives, ovarian cancer, preventive effect

## Abstract

**Background:**

Ovarian cancer ranks fourth in terms of incidence among gynecological malignancies and third as a cause of cancer death in the gynecological field, with oral contraceptives (OCs) exhibiting a potential relationship to ovarian cancer chemoprevention. This meta-analysis aimed to investigate the association between exposure to OCs and the incidence of ovarian cancer.

**Methods:**

Following the PRISMA (Preferred Reporting Items for Systematic Reviews and Meta-Analyses) guidelines, PubMed, Web of Science, Embase, and the Cochrane Library were searched for cohort studies on OCs and ovarian cancer incidence, covering the period from the establishment of each database to May 2025. Hazard ratio (HR) and 95% confidence interval (95% CI) were applied to assess the association between OCs and ovarian cancer incidence, with subgroup analyses performed for duration of use, time since OCs discontinuation, and geographical region.

**Results:**

This meta-analysis included a total of 11 cohort studies. The results revealed that the OCs exposure group had a significantly lower ovarian cancer incidence than the control group (HR: 0.80, 95% CI: 0.71–0.89; P < 0.001; I^2^ = 53.6%). However, no significant difference in incidence was noted between both groups for individuals with an OCs exposure duration of less than 5 years (HR: 0.98, 95% CI: 0.84–1.16; P = 0.857), and a lower incidence was exclusively observed in the OCs exposure group with use lasting more than 5 years (HR: 0.66, 95% CI: 0.58–0.76; P < 0.001). Subgroup analysis revealed that in both European (HR: 0.74, 95% CI: 0.61–0.91; P = 0.004) and American (HR: 0.83, 95% CI: 0.68–1.01) cohorts, the incidence of ovarian cancer in the exposed group was lower than that in the control group. However, the difference in the latter did not reach statistical significance (P = 0.064), while no difference in ovarian cancer incidence was observed between the two groups in the Asian cohort (HR: 0.93, 95% CI: 0.71–1.23; P = 0.628).

**Conclusion:**

This study demonstrated that OCs exposure can exert a preventive effect on ovarian cancer, but such prevention necessitates a duration of use of more than 5 years, and the effect is impacted by geographical factors. In the future, OCs may emerge as one of the potential measures for ovarian cancer prevention.

## Introduction

Ovarian cancer is the fourth most common gynecologic malignancy and the third leading cause of cancer death in women (Li et al., 2025). GLOBOCAN 2022 data indicate over 320000 new ovarian cancer cases and more than 200000 related deaths worldwide (Zhu et al., 2024). Mortality is predicted to increase by 40% by the year 2050 (Xie et al., 2026). Ovarian cancer is divided into three primary groups: epithelial, germ cell, and sex-cord stromal tumors, with epithelial ovarian cancer being the most common clinical form (Devouassoux-Shisheboran and Genestie, 2015). Previous studies have indicated that age, dietary habits, nulliparity, hormone replacement therapy, and family medical history are all risk factors for ovarian cancer (Ali et al., 2023). Due to the lack of specific symptoms and signs (Penny, 2020), ovarian cancer typically presents at an intermediate to advanced stage at the time of consultation (Stewart et al., 2019). It also has an extremely poor prognosis, 15% of women with ovarian cancer died within 2 months of diagnosis (Reid et al., 2021). Thus, the prevention of ovarian cancer assumes great significance.

Existing studies have confirmed that the Mediterranean diet, increased exercise, and a healthy body weight protect against ovarian cancer by balancing hormone levels and alleviating inflammatory reactions (Farina et al., 2025). Oral contraceptives (OCs) use is potentially associated with the prevention of ovarian cancer, possibly through mechanisms including reduced ovulation, induced necrosis and shedding of fallopian tube epithelial cells, and inhibition of inflammatory signaling pathways (Berchuck and Schildkraut, 1997; Wu et al., 2017; Murphy et al., 2010). Nevertheless, prospective cohort studies indicate that no reduction in ovarian cancer risk is detectable among individuals with an OCs exposure duration of fewer than 15 years, whereas a tendency toward decreased risk is observed in those who have used OCs for more than 15 years (Shafrir et al., 2017). Moreover, the efficacy of OCs varies among populations in different geographical regions. In a Singaporean cohort study, no statistically significant association was found between OCs exposure and the incidence of ovarian cancer (Gay et al., 2015). In contrast, a prospective cohort study conducted in the United Kingdom demonstrated that OCs could effectively reduce the incidence of ovarian cancer by 35% (Hippisley-Cox and Coupland, 2015).

In recently published meta-analyses, some articles suggest that exposure to OCs for more than 1 year can reduce the incidence of ovarian cancer (H et al., 2013), while others argue that the preventive effect is only observed after more than 5 years of use (Arshadi et al., 2024), leading to controversies over the optimal effective duration of use. Unfortunately, these articles include case-control studies, with effect sizes expressed as odds ratio or relative risk, which are of lower reference value compared to hazard ratio (HR). Additionally, they lack further subgroup analyses of factors such as age and region. Therefore, this meta-analysis aims to review relevant published cohort studies to explore the relationship between OCs exposure and the incidence of ovarian cancer, with a particular focus on duration of exposure, geographic regions, and time since OCs discontinuation, all of which have been inconsistent or debated in prior studies.

## Methods

### Literature search strategy

This study was designed and conducted in accordance with the Preferred Reporting Items for Systematic Reviews and Meta-Analyses (PRISMA) guidelines (Page et al., 2021) and was registered with the International Prospective Register of Systematic Reviews (PROSPERO identifier: CRD420251063293). Records from the initiation of the four databases (PubMed, Web of Science, Embase, and the Cochrane Library) until May 2025 were included in the literature search scope, which targeted cohort studies examining the link between OCs exposure and ovarian cancer incidence. Additionally, references from published articles were manually retrieved to maintain the comprehensiveness of the included studies. Search terms combined Medical Subject Headings (MeSH) and free text words, primarily included: (oral contraceptives OR combined oral contraceptives OR hormonal oral contraceptive) AND (ovarian cancer OR ovarian neoplasms), with detailed search strategies provided in Supplementary Search Strategy. After removing duplicate articles through EndNote, two reviewers independently performed literature retrieval and screening; any discrepancies were resolved by a third reviewer.

### Inclusion criteria

Inclusion criteria were established according to the PICOS principle (Participants, Intervention, Control, Outcomes, Study design).

1. P: The study included women with no history of ovarian cancer.
2. I: Women in the exposed group used OCs.
3. C: Women in the control group were not exposed to OCs (adjustments were made if other sources of hormonal exposure were present).
4. O: The outcome was the incidence of ovarian cancer.
5. S: The study design was cohort study.

### Exclusion criteria

1. The full text or data of these studies was unavailable.
2. The data could not be statistically analyzed.
3. The article was not published in English.
4. Participants with a history of other gynaecologic malignancies.
5. If the cohort of an article had been updated, the more updated or comprehensive article was selected.

### Data extraction and quality assessment

Two reviewers independently extracted data, and any discrepancies were addressed by a third reviewer. Specific data were extracted in line with a pre-formulated plan, which comprised authors, publication year, country, recruitment period, follow-up period, age, ovarian cancer incidence, and adjustments.

The Newcastle-Ottawa Scale (NOS) was used to assess the quality of cohort studies. The NOS consists of three categories: study population selection, inter-group comparability, and outcome measurement. In detail, it includes eight scoring items, with a full score of 9 and studies with a score of 6 or higher are deemed high-quality. Two assessors independently conducted the quality assessment, and a third assessor made the final decision in case of ambiguities.

### Statistical analysis

Statistical analyses were conducted using Stata 12.0. The association between OCs exposure and ovarian cancer incidence was evaluated using HR with 95% confidence interval (95% CI). When the HR was <1, OCs exposure was indicated to be a protective factor; when the HR was >1, OCs exposure was considered a risk factor. Heterogeneity between studies was examined using the chi-square test, with I^2^ as the measure, categorized as low (I^2^ ≤25%), moderate (25% < I^2^<75%), or high (I^2^ ≥75%) (Higgins et al., 2003). Due to significant heterogeneity across the included studies (attributable to differences in ethnicity, OCs type, lifestyle, and age of participants), a random-effects model was applied for all analyses. Subgroups were pre-specified according to factors including duration of exposure, geographic region, and time since OCs discontinuation (stop taking OCs). If more than 10 studies were included, sensitivity analyses were conducted to ensure the stability of results, and publication bias was evaluated using Begg’s test (Lin et al., 2018). All tests were two-tailed, with statistical significance set at P < 0.05.

The quality of evidence for each outcome was assessed using the GRADEpro GDT software in accordance with the GRADE (Grading of Recommendations Assessment, Development and Evaluation) approach. As all included studies were observational cohort studies, the initial level of evidence was deemed low certainty. The evidence was downgraded based on five domains: risk of bias, inconsistency, indirectness, imprecision, and publication bias. Upgrading was considered for a large treatment effect, a clear dose-response gradient, or plausible residual confounding that would attenuate the observed effect. The overall certainty of evidence was classified into four levels: high, moderate, low, and very low, to reflect the confidence and reliability of the pooled estimates.

## Results

### Retrieval process

**FIGURE 1:**
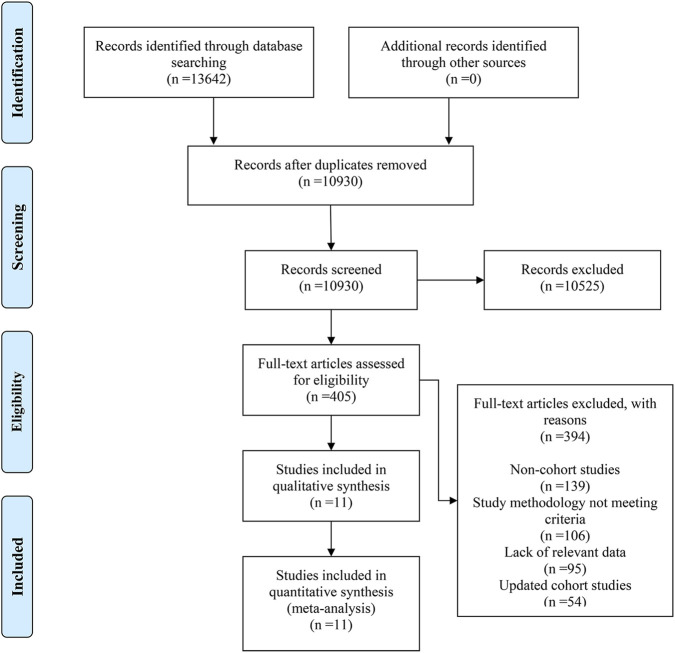
PRISMA 2020 flow diagram illustrating the study selection process.

Up to May 2025, a total of 13642 records were retrieved through searches in four electronic databases—PubMed, Web of Science, Embase, and the Cochrane Library, and no additional records were obtained from other sources. Duplicates were removed with EndNote, and 10930 independent studies were left. Through screening titles and abstracts, 10525 studies were excluded, then 405 studies proceeded to full-text review. After full-text assessment, 394 studies were excluded. Of these, 139 were excluded as they did not belong to cohort studies, 106 were excluded because their research methodologies did not meet the criteria, 95 were excluded due to the lack of relevant data, and 54 were excluded as a result of cohort updates. Finally, a total of 11 studies were included (H et al., 2013; Arshadi et al., 2024; Page et al., 2021; Sar et al., 2020; Laaksonen et al., 2019; McGuire et al., 2016; H et al., 2015; Fathalla, 1971; Peres et al., 2017; Murdoch, 2005; Fortner et al., 2015), and the PRISMA 2020 flow diagram illustrating the study selection process is presented in Figure 1.

### Basic characteristics of the study and quality assessment

These 11 studies were cohort studies published between 2010 and 2021, with recruitment periods ranging from 1986 to 2013 and follow-up periods ranging from 1986 to 2019. Among them, four were conducted in America, two in the United Kingdom, one in the Netherlands, one in Australia, one in Singapore, one in China, and one in Europe, covering women aged 18–106 years and none of the participants had ovarian cancer before enrollment. The types of OCs were mainly compound preparations composed of long-acting estrogens such as quinestrol and progestogens such as norgestrel. The exposed group used OCs, while the control group did not. The main outcome was the incidence of ovarian cancer. The exposure group comprised a total of more than 3.6 million participants, with the control group totaling more than 1.2 million individuals. The median follow-up durations were found to range approximately from 4.9 years to 21 years. Various factors, including age, parity, educational attainment, BMI, family history of cancer, and menopausal status, were adjusted for in the studies. Details are available in Table 1 and Supplementary Table S1.

**TABLE 1:** Characteristics of studies included in the meta-analysis.

Author	Year	Country	Recruitment period	Follow-up period	Age	No. of patients with ovarian cancer	
						Exposure	Control
Karlsson T	2021	Britain	2006–2010	2006–2019	35–80	1,419	539
Bethea T N	2017	America	1995	1995–2013	21–69	80	35
Hippisley-Cox J	2015	Britain	1998–2013	1998–2013	25–84	NA	NA
Braem M G	2010	Netherlands	1986	1986–2002	55–69	65	310
Gay G M	2015	Singapore	1994–1997	1994–2011	50–64	37	70
Sarink D	2020	America	1993–1996	1993–2014	45–75	NA	NA
Laaksonen M A	2019	Australia	1990–2009	1990–2012	18–106	152	110
McGuire V	2016	America	1993–1998	1993–2011	≥50	NA	NA
Shafrir A L	2017	America	1989	1989–2013	25–42	NA	36
Fortner R T	2015	Europe	1992–2000	1992–2010	25–75	530	574
Huang Z	2015	China	1997–2000	1997–2011	40–70	37	137

No., number; NA, not available.

Quality evaluation of the included cohort studies was conducted using the Newcastle-Ottawa Scale (NOS). The final scores fell between 7 and 9, indicating that all were high-quality studies, as detailed in Supplementary Table S2.

### Primary outcome

**FIGURE 2:**
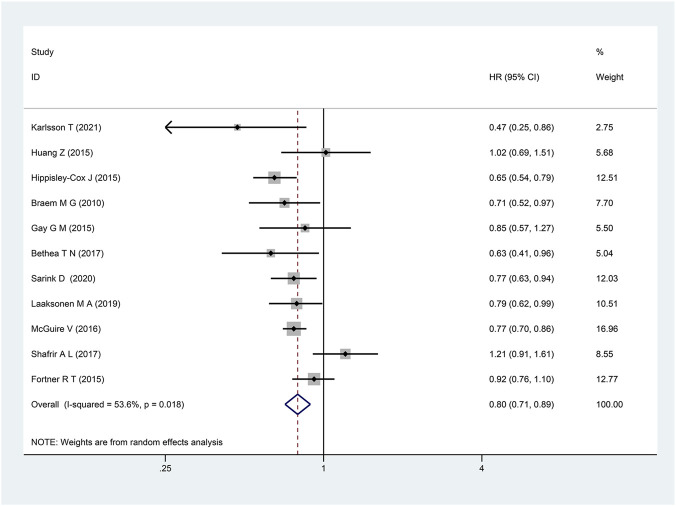
Forest plot showing the association between OCs exposure and the incidence of ovarian cancer (P < 0.001).

The eleven included cohort studies were pooled, and the findings demonstrated that the group exposed to OCs had a lower incidence of ovarian cancer compared with the control group (HR: 0.80, 95% CI: 0.71–0.89; P < 0.001; I^2^ = 53.6%), as illustrated in Figure 2.

**FIGURE 3:**
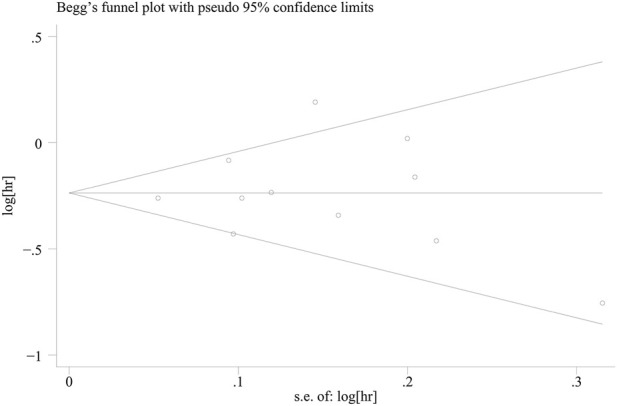
Begg’s publication bias plot in the meta-analysis (P = 0.64).

**FIGURE 4:**
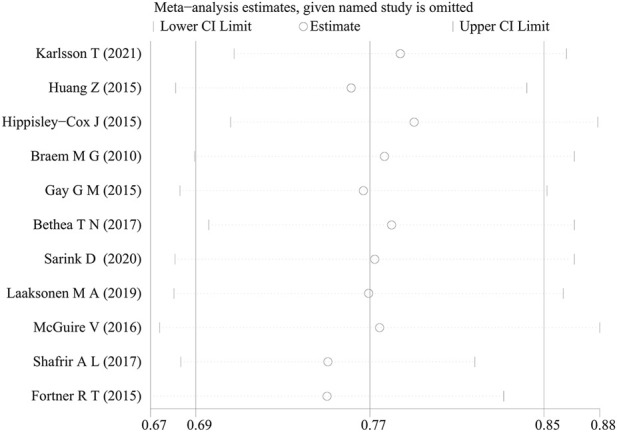
Sensitivity analysis plot in meta-analysis.

Begg’s test was used to assess publication bias among the included studies, and the results indicated that no significant publication bias was observed (P = 0.64), with details provided in Figure 3. Sensitivity analysis was conducted by sequentially excluding each study, and the results remain stable, as shown in Figure 4.

### Subgroup analysis

**FIGURE 5:**
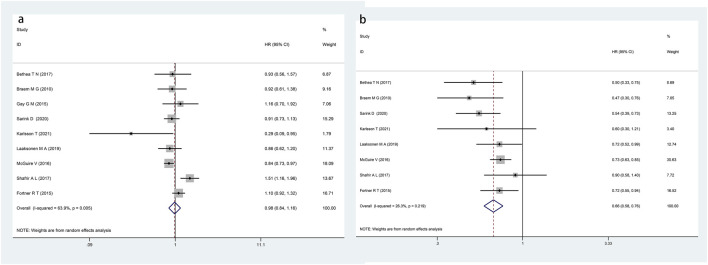
Forest plot showing the association between the duration of oral contraceptive exposure and the incidence of ovarian cancer (**(a)** duration of use <5 years, P = 0.857; **(b)** duration of use >5 years, P < 0.001).

A subgroup analysis was performed based on the duration of OCs exposure, with a total of nine cohort studies included. The results indicated that there was no significant difference in the incidence of ovarian cancer between the exposure group with less than 5 years of usage (cumulative total duration of OCs exposure) and the control group (HR: 0.98, 95% CI: 0.84–1.16; P = 0.857), as detailed in Figure 5a. Of note, after combining eight cohort studies with more than 5 years of usage, the OCs exposure group was found to have a lower ovarian cancer incidence (HR: 0.66, 95% CI: 0.58–0.76; P < 0.001), as depicted in Figure 5b. Stratified by the duration since discontinuation, the exposure group showed a lower incidence of ovarian cancer than the control group (HR: 0.62, 95% CI: 0.52–0.73; P < 0.001). Moreover, a lower incidence was observed in the exposure group versus the control group for both the subgroup with less than 10 years since discontinuation (HR: 0.60, 95% CI: 0.38–0.94; P = 0.026) and the subgroup with more than 10 years since discontinuation (HR: 0.62, 95% CI: 0.52–0.75; P < 0.001), as shown in Table 2.

**TABLE 2:** Subgroup analysis of the incidence of ovarian cancer for OCs exposure compared to non-exposure.

Subgroup	No. of studies	HR	95% CI	P	I (Zhu et al., 2024) (%)
Discontinue taking OCs					
Discontinuation	3	0.62	(0.52, 0.73)	<0.001	0
<10 years	2	0.60	(0.38, 0.94)	0.026	0
≥10 years	2	0.62	(0.52, 0.75)	<0.001	0
Area					
Asia	2	0.93	(0.71, 1.23)	0.628	0
Europe	4	0.74	(0.61, 0.91)	0.004	57.3
America	4	0.83	(0.68, 1.01)	0.064	69.8
African American, OCs use <5 years	2	0.91	(0.62, 1.33)	0.621	0
African American, OCs use ≥5 years	2	0.54	(0.37, 0.76)	0.001	0
Epithelial ovarian cancer	8	0.84	(0.76, 0.94)	0.002	44.7
OCs use <5 years	6	1.00	(0.84, 1.20)	0.987	71.5
OCs use ≥5 years	6	0.68	(0.59, 0.79)	<0.001	28.9

No., number; OCs, oral contraceptives; HR, hazard ratio; CI, confidence interval.

Stratified by region, subgroup analyses were conducted for three regions: Asia, Europe, and the Americas. In two studies involving the Asian population, no significant difference in the incidence of ovarian cancer was observed between the two groups (HR: 0.93, 95% CI: 0.71–1.23; P = 0.628). However, in four European cohorts, the OCs exposure group had a lower incidence (HR: 0.74, 95% CI: 0.61–0.91; P = 0.004). Subgroup analysis of four American population studies indicated a lower incidence in the OCs exposure group versus the control group, but the difference did not reach statistical significance (HR: 0.83, 95% CI: 0.68–1.01; P = 0.064). Among African Americans, OCs exposure <5 years showed no significant difference (HR: 0.91, 95% CI: 0.62–1.33; P = 0.621), but exposure >5 years was associated with a significantly lower incidence (HR: 0.54, 95% CI: 0.37–0.76; P = 0.001), as detailed in Table 2. Eight studies on epithelial ovarian cancer were included. The exposed group had a lower ovarian cancer incidence than the control group (HR: 0.84, 95% CI: 0.76–0.94; P = 0.002). No significant difference was found in the subgroup with <5 years of exposure (HR: 1.00, 95% CI: 0.84–1.20; P = 0.987), whereas the subgroup with ≥5 years of exposure showed a significantly lower incidence (HR: 0.68, 95% CI: 0.59–0.79; P < 0.001). Details are presented in Table 2.

The certainty of evidence for 14 outcomes was assessed using the GRADE approach, and these results are summarized in Supplementary Table S3. Nine outcomes were graded as high certainty, including overall OCs exposure, long-term OCs exposure, discontinuation of OCs, and epithelial ovarian cancer-related outcomes. Four outcomes were moderate certainty, and only OC exposure among American populations was rated as very low certainty. Downgrading was mainly attributed to substantial heterogeneity and imprecision, while a distinct dose-response gradient allowed upgrading for several comparisons. The overall evidence confirmed that long-term OCs exposure is associated with a lower risk of ovarian cancer, as detailed in Supplementary Table S3 (GRADE analysis).

## Discussion

By synthesizing data from 11 cohort studies, this meta-analysis yielded results that aligned with prior meta-analyses in certain aspects, providing evidence that OCs exposure is associated with a decreased incidence of ovarian cancer, and this protective effect remains even after OCs exposure is ceased. A key observation from this study was the significant impact of OCs exposure duration on preventive outcomes: OCs exposure lasting less than 5 years failed to exhibit a preventive effect, while exposure extending beyond 5 years led to a substantial reduction in ovarian cancer incidence. Moreover, the ovarian protective effect of OCs varied by geographical region—European populations experienced the strongest preventive effect, Asian populations showed no such effect, and American populations demonstrated an intermediate level of protection.

OCs, primarily containing synthetic estrogen and progesterone, achieve contraception by inhibiting ovulation, which may contribute to their preventive effect against ovarian cancer. It is generally believed that repeated ovulation contributes to ovarian carcinogenesis. Women with a higher number of ovulations have a 59% higher risk of ovarian cancer than those with fewer ovulations (Peres et al., 2017). Studies have suggested that reactive oxygen species generated during follicular rupture at ovulation may induce DNA damage in ovarian epithelial cells. If ovarian epithelial cells with unrepaired DNA damage survive and proliferate clonally, this may lead to the development of ovarian cancer (Murdoch, 2005). OCs exert a protective effect against ovarian cancer primarily through ovulation inhibition (Bastianelli et al., 2018). Estrogen and progesterone, the core components of these formulations, modulate the hypothalamic-pituitary-ovarian axis: they attenuate hypothalamic secretion of gonadotropin-releasing hormone, suppress the activity of gonadotropin-producing cells in the anterior pituitary (Lobo and Stanczyk, 1994), and eliminate the mid-cycle surges of luteinizing hormone and follicle-stimulating hormone that drive periovulatory follicular maturation (Sondheimer, 2008). This cascade of effects prevents follicular maturation and rupture, thereby achieving ovulation inhibition and reducing the “ovulatory age”—calculated as the time from menarche to ovarian cancer diagnosis or menstrual cessation, minus the “protective time” provided by ovulation-reducing factors like OCs (Casagrande et al., 1979). A longer duration of OCs exposure correlates with a shorter ovulatory age and a more potent preventive effect against ovarian cancer. In line with this, the results of this meta-analysis also confirm that long-term OCs exposure (over 5 years) can effectively prevent ovarian cancer. Additionally, OCs exposure for more than 5 years reduces the total lifetime number of ovulations by roughly 15% (Berchuck and Schildkraut, 1997). In contrast, OCs exposure for less than 5 years is insufficient to prevent ovarian cancer due to the limited reduction in ovulation frequency, and no preventive effect was detected in short-term OCs exposure (less than 5 years) in this study.

Numerous studies have demonstrated that women who experience menarche at an earlier age are more likely to develop ovarian cancer (Lee et al., 2022), and age at menopause is positively correlated with ovarian cancer risk (Reid et al., 2017)—this is largely linked to an elevated total number of lifetime ovulations. Moreover, perimenopausal and postmenopausal women often use hormone replacement therapy (Reid et al., 2017). Animal studies suggest that such therapy can stimulate ovarian epithelial cells and raise the mutation risk (Vuong et al., 2018). As with the proliferation and repair of ovarian epithelial cells during ovulation, this may increase the risk of developing ovarian cancer. All these studies highlight the critical influence of repeated ovulation on ovarian cancer, whereas OCs can provide a favorable preventive effect against ovarian cancer by suppressing ovulation.

A proposed theory suggests that the precursor of ovarian cancer may originate from an occult intraepithelial carcinoma in the fimbrial region of the fallopian tube, referred to as “serous tubal intraepithelial carcinoma (STIC)”, whereas the ovary is merely secondarily involved (Kurman, 2013). Clinical studies indicate that most tumor-specific alterations in ovarian cancer—including those affecting tumor protein p53 (TP53), BRCA1, BRCA2, or PTEN—are present in STICs. This evidence further validates that p53 signatures and STICs act as precursors to ovarian cancer (Labidi-Galy et al., 2017). Guided by this theory, extensive research has validated that salpingectomy can reduce the risk of ovarian cancer by 35%. Moreover, compared with unilateral salpingectomy, bilateral salpingectomy reduces the risk of ovarian cancer by 50%, showing a more significant effect (Darelius et al., 2017; Falconer et al., 2015). This further indicates a close association between the fallopian tubes and ovarian cancer, and OCs can act on the fallopian tubes to prevent ovarian cancer. Progestogens in OCs, acting via progesterone receptors, can induce programmed necrosis and shedding of fallopian tube epithelial cells in TP53-deficient mice through the TNF-α/RIPK1/RIPK3/p-MLKL signaling pathway. This process eliminates fallopian tube epithelial cells with TP53 molecular alterations, preventing their further transformation into cancer cells and thereby achieving the goal of ovarian cancer prevention (Wu et al., 2017). Even after OCs discontinuation, progestogens in the body may continue to exert effects on epithelial cells for a certain period, sustaining this abnormal cell-clearing function and maintaining the preventive effect against ovarian cancer. As demonstrated in this meta-analysis, such protective efficacy endures for over 10 years post-discontinuation.

OCs mitigate inflammatory responses, which in turn contributes to the prevention of ovarian cancer (Murphy et al., 2010). Previous studies have indicated that inflammation is a key driver of ovarian cancer progression. In an animal study, ovarian cancer cells were surgically implanted into the fallopian tubes of mice; it was observed that the cancer cells tended to adhere to sites with inflammation, whereas ovaries without inflammatory changes did not exhibit increased cancer cell seeding (Jia et al., 2018). In humans, the upregulation of the key inflammatory signaling pathway nuclear factor-kappa B (NF-κB) and inflammatory cytokines (including IL-6, TNF-α, and IL-8) promotes the formation of the tumor microenvironment, suppresses anti-tumor immune responses, and facilitates immune evasion (Liu et al., 2025). Progestogens contained in OCs act as strong inhibitors of NF-κB nuclear translocation (Maia et al., 2013), enabling them to block inflammatory signaling cascades. In contrast, estrogens in OCs can inhibit NF-κB activation through the regulation of let-7a and miR-125b (Murphy et al., 2010), which in turn inhibits the development of the tumor microenvironment and exerts a preventive effect against ovarian cancer.

By regulating hormones to suppress ovulation and inhibiting inflammatory signaling pathways, OCs exert a preventive effect against ovarian cancer (Berchuck and Schildkraut, 1997; Murphy et al., 2010). Currently, established protective factors such as the Mediterranean diet, increased exercise, and maintenance of a healthy weight may reduce ovarian cancer risk through the same pathways (Farina et al., 2025), indicating that OCs share essential commonalities with universally acknowledged ovarian cancer prevention strategies.

Geographical factors modulate the preventive effect of OCs exposure on ovarian cancer: the effect is strongest in Europeans, non-existent in Asians, and moderate in Americans. One potential explanation is that Western populations have a higher mutation rate in exon 8 of the TP53 gene than Asian populations. As OCs can trigger apoptosis in TP53-mutated fallopian tube epithelial cells and specifically inhibit cancer cell development, their preventive efficacy is better in European and American populations relative to Asian populations (Dansonka-Mieszkowska et al., 2006). Moreover, compared with Asians, European Caucasians exhibit higher anti-Müllerian hormone levels—a marker indicating more sufficient ovarian reserve in Caucasian women, which suggests they may have a greater total number of lifetime ovulations. Given that OCs reduce ovarian cancer risk by decreasing the number of ovulations, this may also be one of the reasons why OCs exert a better preventive effect in Europeans (Bleil et al., 2014). However, as this study only included two cohorts of the Asian population, the limited sample size will reduce statistical power. In contrast, in the subgroup of the American population, the incidence of ovarian cancer in the OC-exposed group was lower than that in the control group, but the difference did not reach statistical significance—likely due to the inclusion of only four cohort studies of the American population. Only one Australian study was included, which precluded subgroup analyses for the Australian population. Therefore, larger-scale and more extensive studies are required to explore and verify the relationship between the preventive effect of OCs and geographical regions. Notably, in the subgroup analysis of OCs exposure duration in the American population, OCs exposure for less than 5 years showed no preventive effect, while exposure for more than 5 years did. This further supports the aforementioned conclusions. However, other studies have indicated that OCs may increase the risk of breast cancer, with exposure for more than 5 years associated with an approximately 40% elevated risk. Therefore, the decision to use OCs should be made after thorough communication and careful assessment with patients. In addition, further clinical studies are warranted to evaluate the benefits and risks of OCs.

In this study, HR served as the effect measure to conduct a comprehensive analysis of the association between OCs exposure and ovarian cancer incidence, encompassing multiple subgroups including OCs exposure duration, OCs discontinuation time, and geographical location. Of greater significance, this meta-analysis incorporated 11 high-quality cohort studies from the recent decade (2010–2021), which offers clinical significance for elucidating the preventive effect of OCs exposure on ovarian cancer.

Nevertheless, this study is not without limitations. First, the number of included studies in some subgroups was relatively small, which may lead to chance findings in the subgroup analysis. Additionally, the limited number of included studies precluded subgroup analyses according to age, parity, menopausal status, race, and other histological types of ovarian cancer. Second, this meta-analysis was unable to explore the effects of different types and dosages of OCs on the incidence of ovarian cancer. Finally, the “healthy user bias” must be considered: individuals who are exposed to OCs may possess stronger health consciousness and cancer prevention awareness, and thus adhere to a more disciplined lifestyle. Their lower ovarian cancer risk might thus be ascribed to their healthy habits and regular physical examinations, rather than the preventive efficacy of OCs. Furthermore, recall bias, extra hormone exposure, number of deliveries, differences in participants’ dietary patterns, changes in OCs formulations and therapeutic protocols are potential factors accounting for the substantial heterogeneity detected in certain subgroups.

## Conclusion

Findings of this study show that the exposure to OCs can prevent ovarian cancer, and this protective effect persists even after OCs discontinuation. Notably, however, exposure to OCs for more than 5 years is required to reduce the risk of ovarian cancer; no protective effect has been observed with a usage duration of less than 5 years. Therefore, long-term and regular OCs exposure is of great importance. In addition, this study further found that the preventive effect of OCs varies across different regions: it is most effective in European women, least effective in Asian women, and moderate in American women. This association between the preventive effect of OCs and region warrants further investigation. In the future, OCs may emerge as one of the potential measures for ovarian cancer prevention. Finally, due to the limited number of included cohort studies, a more comprehensive subgroup analysis could not be conducted. Thus, larger-scale and additional studies are needed to explore and validate these conclusions.

## Data Availability

The original contributions presented in the study are included in the article/Supplementary Material, further inquiries can be directed to the corresponding author.

## Supplementary material

SUPPLEMENTARY TABLE S1 Additional information about studies included in the meta-analysis.

SUPPLEMENTARY TABLE S2 Quality assessment of the cohort studies included. Supplementary search strategy.

SUPPLEMENTARY TABLE S3 GRADE Rating of the Meta-Analysis.
