# Supplementary material for: Oral contraceptives exposure may reduce the risk of ovarian cancer: a meta-analysis based on cohort studies
Source: Front Pharmacol. 2026 Apr 1;17:1732719. doi: 10.3389/fphar.2026.1732719 (PMC13079376; doi:10.3389/fphar.2026.1732719)
Supplement: Supplementary file 1 [file Table1.docx]

**Supplementary Table 1. Additional information about studies included in the meta-analysis.**

| Author | Year | Center status | Median follow-up | Adjustment |
| --- | --- | --- | --- | --- |
| Karlsson T | 2021 | Multi-center | 11 | age |
| Bethea T N | 2017 | Single-center | NA | age, questionnaire cycle, parity, lactation, age at first birth, age at last birth, hysterectomy, tubal ligation, menopausal status, use of postmenopausal female hormones, educational attainment, BMI |
| Hippisley-Cox J | 2015 | Multi-center | NA | age, BMI, interaction between age and family history |
| Braem M G | 2010 | Single-center | 16.3 | age, parity, duration of OCs use |
| Gay G M | 2015 | Single-center | 17 | age, housing type (1-3 room flat, ≥4 room flat, private or landed property, others), family history of breast cancer |
| Sarink D | 2020 | Multi-center | 21 | baseline age, menopausal status, use of OCs, and number of children, race/ethnicity |
| Laaksonen M A | 2019 | Multi-center | 4.9 | age, educational attainment, body fatness, use of OCs, nulliparity |
| McGuire V | 2016 | Multi-center | NA | birth year, parity |
| Shafrir A L | 2017 | Single-center | 20 | age, calendar time, tubal ligation, parity, breastfeeding duration, missing breast-feeding duration, laparoscopically confirmed endometriosis, BMI |
| Fortner R T | 2015 | Multi-center | 11 | full-term pregnancy, use of OCs, menopausal status, age at menopause, use of HRT |
| Huang Z | 2015 | Single-center | 12.6 | age at recruitment, educational attainment, years of ovulation, irregular ovulatory cycles, first-degree family history of cancer, regular exercise in past 5 years, other contraceptive methods |

NA, not available; BMI, body mass index; OCs, oral contraceptives; HRT, hormone replacement therapy
