# Supplementary material for: Oral contraceptives exposure may reduce the risk of ovarian cancer: a meta-analysis based on cohort studies
Source: Front Pharmacol. 2026 Apr 1;17:1732719. doi: 10.3389/fphar.2026.1732719 (PMC13079376; doi:10.3389/fphar.2026.1732719)
Supplement: Supplementary file 2 [file Table3.docx]

**Author(s):**

**Question:** The association between oral contraceptives (OCs) and the risk of ovarian cancer

**Setting:**

**Bibliography:**

| **Certainty assessment** | | | | | | | **№ of patients** | | **Effect** | | **Certainty** | **Importance** |
| --- | --- | --- | --- | --- | --- | --- | --- | --- | --- | --- | --- | --- |
| **№ of studies** | **Study design** | **Risk of bias** | **Inconsistency** | **Indirectness** | **Imprecision** | **Other considerations** | **OCs Exposure** | **Non-use of OCs** | **Relative (95% CI)** | **Absolute (95% CI)** |  |  |
| **OCs use** | | | | | | | | | | | | |
| 11 | non-randomised studies | not serious | serious^a^ | not serious | not serious | dose response gradient |  |  | **HR 0.80** (0.71 to 0.89) [OCs use] | **-- per 1,000** (from -- to --) | ⨁⨁⨁⨁ High^a^ | CRITICAL |
|  |  |  |  |  |  |  | - | 0.0% |  | **-- per 1,000** (from -- to --) |  |  |
| **OCs use <5 year** | | | | | | | | | | | | |
| 9 | non-randomised studies | not serious | serious^a^ | not serious | serious^b^ | dose response gradient |  |  | **HR 0.98** (0.84 to 1.16) [OCs use <5 year] | **-- per 1,000** (from -- to --) | ⨁⨁⨁◯ Moderate^a,b^ | CRITICAL |
|  |  |  |  |  |  |  | - | 0.0% |  | **-- per 1,000** (from -- to --) |  |  |
| **OCs use ≥5 year** | | | | | | | | | | | | |
| 8 | non-randomised studies | not serious | serious^a^ | not serious | not serious | dose response gradient |  |  | **HR 0.66** (0.58 to 0.76) [OCs use ≥5 year] | **-- per 1,000** (from -- to --) | ⨁⨁⨁⨁ High^a^ | CRITICAL |
|  |  |  |  |  |  |  | - | 0.0% |  | **-- per 1,000** (from -- to --) |  |  |
| **Discontinue taking OCs** | | | | | | | | | | | | |
| 3 | non-randomised studies | not serious | not serious | not serious | not serious | dose response gradient |  |  | **HR 0.62** (0.52 to 0.73) [Discontinue taking OCs] | **-- per 1,000** (from -- to --) | ⨁⨁⨁⨁ High | IMPORTANT |
|  |  |  |  |  |  |  | - | 0.0% |  | **-- per 1,000** (from -- to --) |  |  |
| **Discontinue taking OCs <10 years** | | | | | | | | | | | | |
| 2 | non-randomised studies | not serious | not serious | not serious | not serious | dose response gradient |  |  | **HR 0.60** (0.38 to 0.94) [Discontinue taking OCs <10 years] | **-- per 1,000** (from -- to --) | ⨁⨁⨁⨁ High | IMPORTANT |
|  |  |  |  |  |  |  | - | 0.0% |  | **-- per 1,000** (from -- to --) |  |  |
| **Discontinue taking OCs ≥10 years** | | | | | | | | | | | | |
| 2 | non-randomised studies | not serious | not serious | not serious | not serious | dose response gradient |  |  | **HR 0.62** (0.52 to 0.75) [Discontinue taking OCs ≥10 years] | **-- per 1,000** (from -- to --) | ⨁⨁⨁⨁ High | IMPORTANT |
|  |  |  |  |  |  |  | - | 0.0% |  | **-- per 1,000** (from -- to --) |  |  |
| **Asia** | | | | | | | | | | | | |
| 2 | non-randomised studies | not serious | not serious | not serious | serious^b^ | none |  |  | **HR 0.93** (0.71 to 1.23) [Asia] | **-- per 1,000** (from -- to --) | ⨁⨁⨁◯ Moderate^b^ | IMPORTANT |
|  |  |  |  |  |  |  | - | 0.0% |  | **-- per 1,000** (from -- to --) |  |  |
| **Europe** | | | | | | | | | | | | |
| 4 | non-randomised studies | not serious | serious^a^ | not serious | not serious | none |  |  | **HR 0.74** (0.61 to 0.91) [Europe] | **-- per 1,000** (from -- to --) | ⨁⨁⨁◯ Moderate^a^ | IMPORTANT |
|  |  |  |  |  |  |  | - | 0.0% |  | **-- per 1,000** (from -- to --) |  |  |
| **America** | | | | | | | | | | | | |
| 4 | non-randomised studies | not serious | serious^a^ | not serious | serious^b^ | none |  |  | **HR 0.83** (0.68 to 1.01) [America] | **-- per 1,000** (from -- to --) | ⨁◯◯◯ Very low^a,b^ | IMPORTANT |
|  |  |  |  |  |  |  | - | 0.0% |  | **-- per 1,000** (from -- to --) |  |  |
| **African American, OCs use <5 year** | | | | | | | | | | | | |
| 2 | non-randomised studies | not serious | not serious | not serious | serious^b^ | dose response gradient |  |  | **HR 0.91** (0.62 to 1.33) [African American, OCs use <5 year] | **-- per 1,000** (from -- to --) | ⨁⨁⨁⨁ High^b^ | IMPORTANT |
|  |  |  |  |  |  |  | - | 0.0% |  | **-- per 1,000** (from -- to --) |  |  |
| **African American, OCs use ≥5 year** | | | | | | | | | | | | |
| 2 | non-randomised studies | not serious | not serious | not serious | not serious | dose response gradient |  |  | **HR 0.54** (0.37 to 0.76) [African American, OCs use ≥5 year] | **-- per 1,000** (from -- to --) | ⨁⨁⨁⨁ High | IMPORTANT |
|  |  |  |  |  |  |  | - | 0.0% |  | **-- per 1,000** (from -- to --) |  |  |
| **Epithelial ovarian cancer** | | | | | | | | | | | | |
| 8 | non-randomised studies | not serious | serious^a^ | not serious | not serious | dose response gradient |  |  | **HR 0.84** (0.76 to 0.94) [Epithelial ovarian cancer] | **-- per 1,000** (from -- to --) | ⨁⨁⨁⨁ High^a^ | CRITICAL |
|  |  |  |  |  |  |  | - | 0.0% |  | **-- per 1,000** (from -- to --) |  |  |
| **Epithelial ovarian cancer, OCs use <5 year** | | | | | | | | | | | | |
| 6 | non-randomised studies | not serious | serious^a^ | not serious | serious^b^ | dose response gradient |  |  | **HR 1.00** (0.84 to 1.20) [Epithelial ovarian cancer, OCs use <5 year] | **-- per 1,000** (from -- to --) | ⨁⨁⨁◯ Moderate^a,b^ | CRITICAL |
|  |  |  |  |  |  |  | - | 0.0% |  | **-- per 1,000** (from -- to --) |  |  |
| **Epithelial ovarian cancer, OCs use <5 year** | | | | | | | | | | | | |
| 6 | non-randomised studies | not serious | serious^a^ | not serious | not serious | dose response gradient |  |  | **HR 0.68** (0.59 to 0.79) [Epithelial ovarian cancer, OCs use <5 year] | **-- per 1,000** (from -- to --) | ⨁⨁⨁⨁ High^a^ | CRITICAL |
|  |  |  |  |  |  |  | - | 0.0% |  | **-- per 1,000** (from -- to --) |  |  |

**CI:** confidence interval; **HR:** hazard ratio

#### Explanations

a. Moderate heterogeneity was observed via the chi-square test (25% < I² < 75%).

b. The 95%CI crosses 1.
