# Supplementary material for: Oral contraceptives exposure may reduce the risk of ovarian cancer: a meta-analysis based on cohort studies
Source: Front Pharmacol. 2026 Apr 1;17:1732719. doi: 10.3389/fphar.2026.1732719 (PMC13079376; doi:10.3389/fphar.2026.1732719)
Supplement: Supplementary file 3 [file Table4.docx]

**Supplementary** **Search Strategy.**

**Pubmed**

| Search number | Query | Search Details | Results |
| --- | --- | --- | --- |
| 1 | (((((((((((((((((((((((((((((((((((((Oral Contraceptive) OR (Contraceptive, Oral)) OR (Oral Contraceptives)) OR (Oral Contraceptives, Low-Dose)) OR (Contraceptives, Low-Dose Oral)) OR (Low-Dose Oral Contraceptives)) OR (Low-Dose Oral Contraceptive)) OR (Oral Contraceptives, Low Dose)) OR (Contraceptive, Low-Dose Oral)) OR (Low Dose Oral Contraceptive)) OR (Oral Contraceptive, Low-Dose)) OR (Oral Contraceptives, Phasic)) OR (Contraceptives, Phasic Oral)) OR (Phasic Oral Contraceptives)) OR (Oral Contraceptives, Hormonal)) OR (Contraceptives, Hormonal Oral)) OR (Hormonal Oral Contraceptives)) OR (Contraceptive Agents, Oral, Hormonal)) OR (Hormonal Oral Contraceptive Agents)) OR (Oral Contraceptive Agents, Hormonal)) OR (Hormonal Oral Contraceptive)) OR (Contraceptive, Hormonal Oral)) OR (Oral Contraceptive, Hormonal)) OR (Hormonal Oral Contraceptive Agent)) OR (Contraceptive Agents, Estrogen)) OR (Estrogen Contraceptive Agents)) OR (Combined Oral Contraceptive)) OR (Contraceptive, Combined Oral)) OR (Oral Contraceptive, Combined)) OR (Oral Contraceptives, Combined)) OR (Combined Oral Contraceptives)) OR (Contraceptives, Combined Oral)) OR (Contraceptive Agents, Female, Combined)) OR ((((((((((((((((((((18,19-dinorpregn-4-en-20-yn-3-one, 13-ethyl-17-hydroxy-, (17alpha)-(-)-) OR (D-Norgestrel)) OR (D Norgestrel)) OR (l-Norgestrel)) OR (l Norgestrel)) OR (Norplant)) OR (Microlut)) OR (Capronor)) OR (Mirena)) OR (Cerazet)) OR (duofem)) OR (Norgeston)) OR (NorLevo)) OR (Plan B)) OR (Vikela)) OR (Microval)) OR (Norplant-2)) OR (Norplant2)) OR (Norplant 2)) OR (Levonorgestrel))) OR ((((((((((Norgestrel) OR (18,19-Dinorpregn-4-en-20-yn-3-one, 13-ethyl-17-hydroxy-, (17alpha)-(+-)-)) OR (DL-Norgestrel)) OR (DL Norgestrel)) OR (Neogest)) OR (Ovrette)) OR (Postinor)) OR (Wy-3707)) OR (Wy3707)) OR (Wy 3707))) OR (((((((Chlormadinon Acetate) OR (Pregna-4,6-diene-3,20-dione, 17-(acetyloxy)-6-chloro-)) OR (Neo-Eunomin)) OR (NeoEunomin)) OR (Neo Eunomin)) OR (Chlormadinone)) OR (Chlormadinone Acetate, (9 beta,10 alpha)-Isomer))) OR ((((((((((((((((((((Medroxyprogesterone Acetate) OR (Medroxyprogesterone 17-Acetate)) OR (Medroxyprogesterone 17 Acetate)) OR (Depo-Medroxyprogesterone Acetate)) OR (Depo Medroxyprogesterone Acetate)) OR (6-alpha-Methyl-17alpha-hydroxyprogesterone Acetate)) OR (6 alpha Methyl 17alpha hydroxyprogesterone Acetate)) OR (Depo-Provera)) OR (DepoProvera)) OR (Depo Provera)) OR (Farlutal)) OR (Provera)) OR (Curretab)) OR (Cycrin)) OR (Gestapuran)) OR (Perlutex)) OR (Veramix)) OR (Medroxyprogesterone 17-Acetate, (6 beta)-Isomer)) OR (Medroxyprogesterone 17-Acetate, (6 alpha,17 alpha)-Isomer)) OR (Pregn-4-ene-3,20-dione, 17-(acetyloxy)-6-methyl-, (6alpha)-))) | "contraceptives oral"[Supplementary Concept] OR "contraceptives oral"[All Fields] OR "oral contraceptive"[All Fields] OR "contraceptives, oral"[MeSH Terms] OR ("contraceptives"[All Fields] AND "oral"[All Fields]) OR "oral contraceptives"[All Fields] OR ("oral"[All Fields] AND "contraceptive"[All Fields]) OR ("contraceptives oral"[Supplementary Concept] OR "contraceptives oral"[All Fields] OR "contraceptive oral"[All Fields] OR "contraceptives, oral"[MeSH Terms] OR ("contraceptives"[All Fields] AND "oral"[All Fields]) OR "oral contraceptives"[All Fields] OR ("contraceptive"[All Fields] AND "oral"[All Fields])) OR ("contraceptives oral"[Supplementary Concept] OR "contraceptives oral"[All Fields] OR "oral contraceptives"[All Fields] OR "contraceptives, oral"[MeSH Terms] OR ("contraceptives"[All Fields] AND "oral"[All Fields]) OR ("oral"[All Fields] AND "contraceptives"[All Fields])) OR ("contraceptives oral"[Supplementary Concept] OR "contraceptives oral"[All Fields] OR "oral contraceptives low dose"[All Fields] OR "contraceptives, oral"[MeSH Terms] OR ("contraceptives"[All Fields] AND "oral"[All Fields]) OR "oral contraceptives"[All Fields] OR ("oral"[All Fields] AND "contraceptives"[All Fields] AND "low"[All Fields] AND "dose"[All Fields])) OR ("contraceptives oral"[Supplementary Concept] OR "contraceptives oral"[All Fields] OR "contraceptives, oral"[MeSH Terms] OR ("contraceptives"[All Fields] AND "oral"[All Fields]) OR "oral contraceptives"[All Fields] OR ("contraceptives"[All Fields] AND "low"[All Fields] AND "dose"[All Fields] AND "oral"[All Fields])) OR ("contraceptives oral"[Supplementary Concept] OR "contraceptives oral"[All Fields] OR "low dose oral contraceptives"[All Fields] OR "contraceptives, oral"[MeSH Terms] OR ("contraceptives"[All Fields] AND "oral"[All Fields]) OR "oral contraceptives"[All Fields] OR ("low"[All Fields] AND "dose"[All Fields] AND "oral"[All Fields] AND "contraceptives"[All Fields])) OR ("contraceptives oral"[Supplementary Concept] OR "contraceptives oral"[All Fields] OR "low dose oral contraceptive"[All Fields] OR "contraceptives, oral"[MeSH Terms] OR ("contraceptives"[All Fields] AND "oral"[All Fields]) OR "oral contraceptives"[All Fields] OR ("low"[All Fields] AND "dose"[All Fields] AND "oral"[All Fields] AND "contraceptive"[All Fields])) OR ("contraceptives oral"[Supplementary Concept] OR "contraceptives oral"[All Fields] OR "oral contraceptives low dose"[All Fields] OR "contraceptives, oral"[MeSH Terms] OR ("contraceptives"[All Fields] AND "oral"[All Fields]) OR "oral contraceptives"[All Fields] OR ("oral"[All Fields] AND "contraceptives"[All Fields] AND "low"[All Fields] AND "dose"[All Fields])) OR ("contraceptives oral"[Supplementary Concept] OR "contraceptives oral"[All Fields] OR "contraceptives, oral"[MeSH Terms] OR ("contraceptives"[All Fields] AND "oral"[All Fields]) OR "oral contraceptives"[All Fields] OR ("contraceptive"[All Fields] AND "low"[All Fields] AND "dose"[All Fields] AND "oral"[All Fields])) OR ("contraceptives oral"[Supplementary Concept] OR "contraceptives oral"[All Fields] OR "low dose oral contraceptive"[All Fields] OR "contraceptives, oral"[MeSH Terms] OR ("contraceptives"[All Fields] AND "oral"[All Fields]) OR "oral contraceptives"[All Fields] OR ("low"[All Fields] AND "dose"[All Fields] AND "oral"[All Fields] AND "contraceptive"[All Fields])) OR ("contraceptives oral"[Supplementary Concept] OR "contraceptives oral"[All Fields] OR "oral contraceptive low dose"[All Fields] OR "contraceptives, oral"[MeSH Terms] OR ("contraceptives"[All Fields] AND "oral"[All Fields]) OR "oral contraceptives"[All Fields] OR ("oral"[All Fields] AND "contraceptive"[All Fields] AND "low"[All Fields] AND "dose"[All Fields])) OR ("contraceptives oral"[Supplementary Concept] OR "contraceptives oral"[All Fields] OR "oral contraceptives phasic"[All Fields] OR "contraceptives, oral"[MeSH Terms] OR ("contraceptives"[All Fields] AND "oral"[All Fields]) OR "oral contraceptives"[All Fields] OR ("oral"[All Fields] AND "contraceptives"[All Fields] AND "phasic"[All Fields])) OR ("contraceptives oral"[Supplementary Concept] OR "contraceptives oral"[All Fields] OR "contraceptives, oral"[MeSH Terms] OR ("contraceptives"[All Fields] AND "oral"[All Fields]) OR "oral contraceptives"[All Fields] OR ("contraceptives"[All Fields] AND "phasic"[All Fields] AND "oral"[All Fields])) OR ("contraceptives oral"[Supplementary Concept] OR "contraceptives oral"[All Fields] OR "phasic oral contraceptives"[All Fields] OR "contraceptives, oral"[MeSH Terms] OR ("contraceptives"[All Fields] AND "oral"[All Fields]) OR "oral contraceptives"[All Fields] OR ("phasic"[All Fields] AND "oral"[All Fields] AND "contraceptives"[All Fields])) OR ("contraceptives oral hormonal"[Pharmacological Action] OR "contraceptives oral hormonal"[Supplementary Concept] OR "contraceptives oral hormonal"[All Fields] OR "oral contraceptives hormonal"[All Fields] OR "contraceptives, oral, hormonal"[MeSH Terms] OR ("contraceptives"[All Fields] AND "oral"[All Fields] AND "hormonal"[All Fields]) OR "hormonal oral contraceptives"[All Fields] OR ("oral"[All Fields] AND "contraceptives"[All Fields] AND "hormonal"[All Fields])) OR ("contraceptives oral hormonal"[Pharmacological Action] OR "contraceptives oral hormonal"[Supplementary Concept] OR "contraceptives oral hormonal"[All Fields] OR "contraceptives, oral, hormonal"[MeSH Terms] OR ("contraceptives"[All Fields] AND "oral"[All Fields] AND "hormonal"[All Fields]) OR "hormonal oral contraceptives"[All Fields] OR ("contraceptives"[All Fields] AND "hormonal"[All Fields] AND "oral"[All Fields])) OR ("contraceptives oral hormonal"[Pharmacological Action] OR "contraceptives oral hormonal"[Supplementary Concept] OR "contraceptives oral hormonal"[All Fields] OR "hormonal oral contraceptives"[All Fields] OR "contraceptives, oral, hormonal"[MeSH Terms] OR ("contraceptives"[All Fields] AND "oral"[All Fields] AND "hormonal"[All Fields]) OR ("hormonal"[All Fields] AND "oral"[All Fields] AND "contraceptives"[All Fields])) OR ("contraceptives oral hormonal"[Pharmacological Action] OR "contraceptives oral hormonal"[Supplementary Concept] OR "contraceptives oral hormonal"[All Fields] OR "contraceptive agents oral hormonal"[All Fields] OR "contraceptives, oral, hormonal"[MeSH Terms] OR ("contraceptives"[All Fields] AND "oral"[All Fields] AND "hormonal"[All Fields]) OR "hormonal oral contraceptives"[All Fields] OR ("contraceptive"[All Fields] AND "agents"[All Fields] AND "oral"[All Fields] AND "hormonal"[All Fields])) OR ("contraceptives oral hormonal"[Pharmacological Action] OR "contraceptives oral hormonal"[Supplementary Concept] OR "contraceptives oral hormonal"[All Fields] OR "hormonal oral contraceptive agents"[All Fields] OR "contraceptives, oral, hormonal"[MeSH Terms] OR ("contraceptives"[All Fields] AND "oral"[All Fields] AND "hormonal"[All Fields]) OR "hormonal oral contraceptives"[All Fields] OR ("hormonal"[All Fields] AND "oral"[All Fields] AND "contraceptive"[All Fields] AND "agents"[All Fields])) OR ("contraceptives oral hormonal"[Pharmacological Action] OR "contraceptives oral hormonal"[Supplementary Concept] OR "contraceptives oral hormonal"[All Fields] OR "contraceptives, oral, hormonal"[MeSH Terms] OR ("contraceptives"[All Fields] AND "oral"[All Fields] AND "hormonal"[All Fields]) OR "hormonal oral contraceptives"[All Fields] OR ("oral"[All Fields] AND "contraceptive"[All Fields] AND "agents"[All Fields] AND "hormonal"[All Fields])) OR ("contraceptives oral hormonal"[Pharmacological Action] OR "contraceptives oral hormonal"[Supplementary Concept] OR "contraceptives oral hormonal"[All Fields] OR "hormonal oral contraceptive"[All Fields] OR "contraceptives, oral, hormonal"[MeSH Terms] OR ("contraceptives"[All Fields] AND "oral"[All Fields] AND "hormonal"[All Fields]) OR "hormonal oral contraceptives"[All Fields] OR ("hormonal"[All Fields] AND "oral"[All Fields] AND "contraceptive"[All Fields])) OR ("contraceptives oral hormonal"[Pharmacological Action] OR "contraceptives oral hormonal"[Supplementary Concept] OR "contraceptives oral hormonal"[All Fields] OR "contraceptives, oral, hormonal"[MeSH Terms] OR ("contraceptives"[All Fields] AND "oral"[All Fields] AND "hormonal"[All Fields]) OR "hormonal oral contraceptives"[All Fields] OR ("contraceptive"[All Fields] AND "hormonal"[All Fields] AND "oral"[All Fields])) OR ("contraceptives oral hormonal"[Pharmacological Action] OR "contraceptives oral hormonal"[Supplementary Concept] OR "contraceptives oral hormonal"[All Fields] OR "oral contraceptive hormonal"[All Fields] OR "contraceptives, oral, hormonal"[MeSH Terms] OR ("contraceptives"[All Fields] AND "oral"[All Fields] AND "hormonal"[All Fields]) OR "hormonal oral contraceptives"[All Fields] OR ("oral"[All Fields] AND "contraceptive"[All Fields] AND "hormonal"[All Fields])) OR ("contraceptives oral hormonal"[Pharmacological Action] OR "contraceptives oral hormonal"[Supplementary Concept] OR "contraceptives oral hormonal"[All Fields] OR "contraceptives, oral, hormonal"[MeSH Terms] OR ("contraceptives"[All Fields] AND "oral"[All Fields] AND "hormonal"[All Fields]) OR "hormonal oral contraceptives"[All Fields] OR ("hormonal"[All Fields] AND "oral"[All Fields] AND "contraceptive"[All Fields] AND "agent"[All Fields])) OR ("contraceptives oral hormonal"[Pharmacological Action] OR "contraceptives oral hormonal"[Supplementary Concept] OR "contraceptives oral hormonal"[All Fields] OR "contraceptive agents estrogen"[All Fields] OR "contraceptives, oral, hormonal"[MeSH Terms] OR ("contraceptives"[All Fields] AND "oral"[All Fields] AND "hormonal"[All Fields]) OR "hormonal oral contraceptives"[All Fields] OR ("contraceptive"[All Fields] AND "agents"[All Fields] AND "estrogen"[All Fields])) OR ("contraceptives oral hormonal"[Pharmacological Action] OR "contraceptives oral hormonal"[Supplementary Concept] OR "contraceptives oral hormonal"[All Fields] OR "contraceptives, oral, hormonal"[MeSH Terms] OR ("contraceptives"[All Fields] AND "oral"[All Fields] AND "hormonal"[All Fields]) OR "hormonal oral contraceptives"[All Fields] OR ("estrogen"[All Fields] AND "contraceptive"[All Fields] AND "agents"[All Fields])) OR ("contraceptives oral combined"[Pharmacological Action] OR "contraceptives oral combined"[Supplementary Concept] OR "contraceptives oral combined"[All Fields] OR "combined oral contraceptive"[All Fields] OR "contraceptives, oral, combined"[MeSH Terms] OR ("contraceptives"[All Fields] AND "oral"[All Fields] AND "combined"[All Fields]) OR "combined oral contraceptives"[All Fields] OR ("combined"[All Fields] AND "oral"[All Fields] AND "contraceptive"[All Fields])) OR ("contraceptives oral combined"[Pharmacological Action] OR "contraceptives oral combined"[Supplementary Concept] OR "contraceptives oral combined"[All Fields] OR "contraceptive combined oral"[All Fields] OR "contraceptives, oral, combined"[MeSH Terms] OR ("contraceptives"[All Fields] AND "oral"[All Fields] AND "combined"[All Fields]) OR "combined oral contraceptives"[All Fields] OR ("contraceptive"[All Fields] AND "combined"[All Fields] AND "oral"[All Fields])) OR ("contraceptives oral combined"[Pharmacological Action] OR "contraceptives oral combined"[Supplementary Concept] OR "contraceptives oral combined"[All Fields] OR "oral contraceptive combined"[All Fields] OR "contraceptives, oral, combined"[MeSH Terms] OR ("contraceptives"[All Fields] AND "oral"[All Fields] AND "combined"[All Fields]) OR "combined oral contraceptives"[All Fields] OR ("oral"[All Fields] AND "contraceptive"[All Fields] AND "combined"[All Fields])) OR ("contraceptives oral combined"[Pharmacological Action] OR "contraceptives oral combined"[Supplementary Concept] OR "contraceptives oral combined"[All Fields] OR "oral contraceptives combined"[All Fields] OR "contraceptives, oral, combined"[MeSH Terms] OR ("contraceptives"[All Fields] AND "oral"[All Fields] AND "combined"[All Fields]) OR "combined oral contraceptives"[All Fields] OR ("oral"[All Fields] AND "contraceptives"[All Fields] AND "combined"[All Fields])) OR ("contraceptives oral combined"[Pharmacological Action] OR "contraceptives oral combined"[Supplementary Concept] OR "contraceptives oral combined"[All Fields] OR "combined oral contraceptives"[All Fields] OR "contraceptives, oral, combined"[MeSH Terms] OR ("contraceptives"[All Fields] AND "oral"[All Fields] AND "combined"[All Fields]) OR ("combined"[All Fields] AND "oral"[All Fields] AND "contraceptives"[All Fields])) OR ("contraceptives oral combined"[Pharmacological Action] OR "contraceptives oral combined"[Supplementary Concept] OR "contraceptives oral combined"[All Fields] OR "contraceptives combined oral"[All Fields] OR "contraceptives, oral, combined"[MeSH Terms] OR ("contraceptives"[All Fields] AND "oral"[All Fields] AND "combined"[All Fields]) OR "combined oral contraceptives"[All Fields] OR ("contraceptives"[All Fields] AND "combined"[All Fields] AND "oral"[All Fields])) OR ("contraceptives oral combined"[Pharmacological Action] OR "contraceptives oral combined"[Supplementary Concept] OR "contraceptives oral combined"[All Fields] OR "contraceptives, oral, combined"[MeSH Terms] OR ("contraceptives"[All Fields] AND "oral"[All Fields] AND "combined"[All Fields]) OR "combined oral contraceptives"[All Fields] OR ("contraceptive"[All Fields] AND "agents"[All Fields] AND "female"[All Fields] AND "combined"[All Fields])) OR ((("18 19 dinorpregn 4 en 20 yn 3 one"[All Fields] AND "13 ethyl 17 hydroxy"[All Fields]) AND "17alpha"[All Fields]) OR ("levonorgestrel"[Supplementary Concept] OR "levonorgestrel"[All Fields] OR "d norgestrel"[All Fields] OR "levonorgestrel"[MeSH Terms]) OR ("levonorgestrel"[Supplementary Concept] OR "levonorgestrel"[All Fields] OR "d norgestrel"[All Fields] OR "levonorgestrel"[MeSH Terms]) OR ("levonorgestrel"[Supplementary Concept] OR "levonorgestrel"[All Fields] OR "l norgestrel"[All Fields] OR "levonorgestrel"[MeSH Terms]) OR ("levonorgestrel"[Supplementary Concept] OR "levonorgestrel"[All Fields] OR "l norgestrel"[All Fields] OR "levonorgestrel"[MeSH Terms]) OR ("levonorgestrel"[Supplementary Concept] OR "levonorgestrel"[All Fields] OR "norplant"[All Fields] OR "levonorgestrel"[MeSH Terms]) OR ("levonorgestrel"[Supplementary Concept] OR "levonorgestrel"[All Fields] OR "microlut"[All Fields] OR "levonorgestrel"[MeSH Terms]) OR ("levonorgestrel"[Supplementary Concept] OR "levonorgestrel"[All Fields] OR "capronor"[All Fields] OR "levonorgestrel"[MeSH Terms]) OR ("levonorgestrel"[Supplementary Concept] OR "levonorgestrel"[All Fields] OR "levonorgestrel"[MeSH Terms] OR "mirena"[All Fields]) OR ("levonorgestrel"[Supplementary Concept] OR "levonorgestrel"[All Fields] OR "cerazet"[All Fields] OR "levonorgestrel"[MeSH Terms]) OR ("levonorgestrel"[Supplementary Concept] OR "levonorgestrel"[All Fields] OR "levonorgestrel"[MeSH Terms]) OR ("levonorgestrel"[Supplementary Concept] OR "levonorgestrel"[All Fields] OR "norgeston"[All Fields] OR "levonorgestrel"[MeSH Terms]) OR ("levonorgestrel"[Supplementary Concept] OR "levonorgestrel"[All Fields] OR "norlevo"[All Fields] OR "levonorgestrel"[MeSH Terms]) OR ("levonorgestrel"[Supplementary Concept] OR "levonorgestrel"[All Fields] OR "plan b"[All Fields] OR "levonorgestrel"[MeSH Terms]) OR ("levonorgestrel"[Supplementary Concept] OR "levonorgestrel"[All Fields] OR "vikela"[All Fields] OR "levonorgestrel"[MeSH Terms]) OR ("levonorgestrel"[Supplementary Concept] OR "levonorgestrel"[All Fields] OR "microval"[All Fields] OR "levonorgestrel"[MeSH Terms]) OR ("levonorgestrel"[Supplementary Concept] OR "levonorgestrel"[All Fields] OR "norplant 2"[All Fields] OR "levonorgestrel"[MeSH Terms]) OR ("levonorgestrel"[Supplementary Concept] OR "levonorgestrel"[All Fields] OR "norplant2"[All Fields] OR "levonorgestrel"[MeSH Terms]) OR ("levonorgestrel"[Supplementary Concept] OR "levonorgestrel"[All Fields] OR "norplant 2"[All Fields] OR "levonorgestrel"[MeSH Terms]) OR ("levonorgestrel"[Supplementary Concept] OR "levonorgestrel"[All Fields] OR "levonorgestrel"[MeSH Terms])) OR ("norgestrel"[Supplementary Concept] OR "norgestrel"[All Fields] OR "norgestrel"[MeSH Terms] OR (("18 19 dinorpregn 4 en 20 yn 3 one"[All Fields] AND "13 ethyl 17 hydroxy"[All Fields]) AND "17alpha"[All Fields]) OR ("norgestrel"[Supplementary Concept] OR "norgestrel"[All Fields] OR "dl norgestrel"[All Fields] OR "norgestrel"[MeSH Terms] OR ("dl"[All Fields] AND "norgestrel"[All Fields])) OR ("norgestrel"[Supplementary Concept] OR "norgestrel"[All Fields] OR "dl norgestrel"[All Fields] OR "norgestrel"[MeSH Terms] OR ("dl"[All Fields] AND "norgestrel"[All Fields])) OR ("norgestrel"[Supplementary Concept] OR "norgestrel"[All Fields] OR "neogest"[All Fields] OR "norgestrel"[MeSH Terms]) OR ("norgestrel"[Supplementary Concept] OR "norgestrel"[All Fields] OR "ovrette"[All Fields] OR "norgestrel"[MeSH Terms]) OR ("norgestrel"[Supplementary Concept] OR "norgestrel"[All Fields] OR "postinor"[All Fields] OR "norgestrel"[MeSH Terms]) OR ("norgestrel"[Supplementary Concept] OR "norgestrel"[All Fields] OR "wy 3707"[All Fields] OR "norgestrel"[MeSH Terms]) OR ("norgestrel"[Supplementary Concept] OR "norgestrel"[All Fields] OR "wy3707"[All Fields] OR "norgestrel"[MeSH Terms]) OR ("norgestrel"[Supplementary Concept] OR "norgestrel"[All Fields] OR "wy 3707"[All Fields] OR "norgestrel"[MeSH Terms])) OR ("chlormadinone acetate"[Supplementary Concept] OR "chlormadinone acetate"[All Fields] OR "chlormadinon acetate"[All Fields] OR "chlormadinone acetate"[MeSH Terms] OR ("chlormadinone"[All Fields] AND "acetate"[All Fields]) OR ("chlormadinon"[All Fields] AND "acetate"[All Fields]) OR ((("pregna 4 6 diene 3 20 dione"[All Fields] AND "17"[All Fields]) AND "acetyloxy"[All Fields]) AND "6 chloro"[All Fields]) OR ("chlormadinone acetate"[Supplementary Concept] OR "chlormadinone acetate"[All Fields] OR "neo eunomin"[All Fields] OR "chlormadinone acetate"[MeSH Terms] OR ("chlormadinone"[All Fields] AND "acetate"[All Fields]) OR ("neo"[All Fields] AND "eunomin"[All Fields])) OR ("chlormadinone acetate"[Supplementary Concept] OR "chlormadinone acetate"[All Fields] OR "neoeunomin"[All Fields] OR "chlormadinone acetate"[MeSH Terms] OR ("chlormadinone"[All Fields] AND "acetate"[All Fields])) OR ("chlormadinone acetate"[Supplementary Concept] OR "chlormadinone acetate"[All Fields] OR "neo eunomin"[All Fields] OR "chlormadinone acetate"[MeSH Terms] OR ("chlormadinone"[All Fields] AND "acetate"[All Fields]) OR ("neo"[All Fields] AND "eunomin"[All Fields])) OR ("chlormadinon"[All Fields] OR "chlormadinone acetate"[Supplementary Concept] OR "chlormadinone acetate"[All Fields] OR "chlormadinone"[All Fields] OR "chlormadinone acetate"[MeSH Terms] OR ("chlormadinone"[All Fields] AND "acetate"[All Fields])) OR ((("chlormadinone acetate"[Supplementary Concept] OR "chlormadinone acetate"[All Fields] OR "chlormadinone acetate"[MeSH Terms] OR ("chlormadinone"[All Fields] AND "acetate"[All Fields])) AND ("9"[All Fields] AND "beta 10"[All Fields] AND ("alpha"[All Fields] OR "alpha s"[All Fields] OR "alphas"[All Fields]))) AND ("isomerism"[MeSH Terms] OR "isomerism"[All Fields] OR "isomer"[All Fields] OR "isomers"[All Fields]))) OR ("medroxyprogesterone acetate"[Supplementary Concept] OR "medroxyprogesterone acetate"[All Fields] OR "medroxyprogesterone acetate"[MeSH Terms] OR ("medroxyprogesterone"[All Fields] AND "acetate"[All Fields]) OR ("medroxyprogesterone acetate"[Supplementary Concept] OR "medroxyprogesterone acetate"[All Fields] OR "medroxyprogesterone 17 acetate"[All Fields] OR "medroxyprogesterone acetate"[MeSH Terms] OR ("medroxyprogesterone"[All Fields] AND "acetate"[All Fields])) OR ("medroxyprogesterone acetate"[Supplementary Concept] OR "medroxyprogesterone acetate"[All Fields] OR "medroxyprogesterone 17 acetate"[All Fields] OR "medroxyprogesterone acetate"[MeSH Terms] OR ("medroxyprogesterone"[All Fields] AND "acetate"[All Fields])) OR ("medroxyprogesterone acetate"[Supplementary Concept] OR "medroxyprogesterone acetate"[All Fields] OR "depo medroxyprogesterone acetate"[All Fields] OR "medroxyprogesterone acetate"[MeSH Terms] OR ("medroxyprogesterone"[All Fields] AND "acetate"[All Fields]) OR ("depo"[All Fields] AND "medroxyprogesterone"[All Fields] AND "acetate"[All Fields])) OR ("medroxyprogesterone acetate"[Supplementary Concept] OR "medroxyprogesterone acetate"[All Fields] OR "depo medroxyprogesterone acetate"[All Fields] OR "medroxyprogesterone acetate"[MeSH Terms] OR ("medroxyprogesterone"[All Fields] AND "acetate"[All Fields]) OR ("depo"[All Fields] AND "medroxyprogesterone"[All Fields] AND "acetate"[All Fields])) OR ("medroxyprogesterone acetate"[Supplementary Concept] OR "medroxyprogesterone acetate"[All Fields] OR "6 alpha methyl 17alpha hydroxyprogesterone acetate"[All Fields] OR "medroxyprogesterone acetate"[MeSH Terms] OR ("medroxyprogesterone"[All Fields] AND "acetate"[All Fields])) OR ("medroxyprogesterone acetate"[Supplementary Concept] OR "medroxyprogesterone acetate"[All Fields] OR "6 alpha methyl 17alpha hydroxyprogesterone acetate"[All Fields] OR "medroxyprogesterone acetate"[MeSH Terms] OR ("medroxyprogesterone"[All Fields] AND "acetate"[All Fields])) OR ("medroxyprogesteron"[All Fields] OR "medroxyprogesterone acetate"[Supplementary Concept] OR "medroxyprogesterone acetate"[All Fields] OR "depo provera"[All Fields] OR "medroxyprogesterone acetate"[MeSH Terms] OR ("medroxyprogesterone"[All Fields] AND "acetate"[All Fields]) OR ("depo"[All Fields] AND "provera"[All Fields]) OR "medroxyprogesterone"[Supplementary Concept] OR "medroxyprogesterone"[All Fields] OR "medroxyprogesterone"[MeSH Terms]) OR ("medroxyprogesterone acetate"[Supplementary Concept] OR "medroxyprogesterone acetate"[All Fields] OR "depoprovera"[All Fields] OR "medroxyprogesterone acetate"[MeSH Terms] OR ("medroxyprogesterone"[All Fields] AND "acetate"[All Fields])) OR ("medroxyprogesteron"[All Fields] OR "medroxyprogesterone acetate"[Supplementary Concept] OR "medroxyprogesterone acetate"[All Fields] OR "depo provera"[All Fields] OR "medroxyprogesterone acetate"[MeSH Terms] OR ("medroxyprogesterone"[All Fields] AND "acetate"[All Fields]) OR ("depo"[All Fields] AND "provera"[All Fields]) OR "medroxyprogesterone"[Supplementary Concept] OR "medroxyprogesterone"[All Fields] OR "medroxyprogesterone"[MeSH Terms]) OR ("medroxyprogesterone acetate"[Supplementary Concept] OR "medroxyprogesterone acetate"[All Fields] OR "farlutal"[All Fields] OR "medroxyprogesterone acetate"[MeSH Terms] OR ("medroxyprogesterone"[All Fields] AND "acetate"[All Fields])) OR ("medroxyprogesteron"[All Fields] OR "medroxyprogesterone acetate"[Supplementary Concept] OR "medroxyprogesterone acetate"[All Fields] OR "provera"[All Fields] OR "medroxyprogesterone acetate"[MeSH Terms] OR ("medroxyprogesterone"[All Fields] AND "acetate"[All Fields]) OR "medroxyprogesterone"[Supplementary Concept] OR "medroxyprogesterone"[All Fields] OR "medroxyprogesterone"[MeSH Terms]) OR ("medroxyprogesterone acetate"[Supplementary Concept] OR "medroxyprogesterone acetate"[All Fields] OR "curretab"[All Fields] OR "medroxyprogesterone acetate"[MeSH Terms] OR ("medroxyprogesterone"[All Fields] AND "acetate"[All Fields])) OR ("medroxyprogesterone acetate"[Supplementary Concept] OR "medroxyprogesterone acetate"[All Fields] OR "cycrin"[All Fields] OR "medroxyprogesterone acetate"[MeSH Terms] OR ("medroxyprogesterone"[All Fields] AND "acetate"[All Fields])) OR ("medroxyprogesterone acetate"[Supplementary Concept] OR "medroxyprogesterone acetate"[All Fields] OR "gestapuran"[All Fields] OR "medroxyprogesterone acetate"[MeSH Terms] OR ("medroxyprogesterone"[All Fields] AND "acetate"[All Fields])) OR ("medroxyprogesterone acetate"[Supplementary Concept] OR "medroxyprogesterone acetate"[All Fields] OR "perlutex"[All Fields] OR "medroxyprogesterone acetate"[MeSH Terms] OR ("medroxyprogesterone"[All Fields] AND "acetate"[All Fields])) OR ("medroxyprogesterone acetate"[Supplementary Concept] OR "medroxyprogesterone acetate"[All Fields] OR "veramix"[All Fields] OR "medroxyprogesterone acetate"[MeSH Terms] OR ("medroxyprogesterone"[All Fields] AND "acetate"[All Fields])) OR ((("medroxyprogesterone acetate"[Supplementary Concept] OR "medroxyprogesterone acetate"[All Fields] OR "medroxyprogesterone 17 acetate"[All Fields] OR "medroxyprogesterone acetate"[MeSH Terms] OR ("medroxyprogesterone"[All Fields] AND "acetate"[All Fields])) AND ("6"[All Fields] AND ("beta"[Journal] OR "beta"[All Fields]))) AND ("isomerism"[MeSH Terms] OR "isomerism"[All Fields] OR "isomer"[All Fields] OR "isomers"[All Fields])) OR ((("medroxyprogesterone acetate"[Supplementary Concept] OR "medroxyprogesterone acetate"[All Fields] OR "medroxyprogesterone 17 acetate"[All Fields] OR "medroxyprogesterone acetate"[MeSH Terms] OR ("medroxyprogesterone"[All Fields] AND "acetate"[All Fields])) AND ("6"[All Fields] AND "alpha 17"[All Fields] AND ("alpha"[All Fields] OR "alpha s"[All Fields] OR "alphas"[All Fields]))) AND ("isomerism"[MeSH Terms] OR "isomerism"[All Fields] OR "isomer"[All Fields] OR "isomers"[All Fields])) OR ((((("progesterone"[Supplementary Concept] OR "progesterone"[All Fields] OR "pregn 4 ene 3 20 dione"[All Fields] OR "progesterone"[MeSH Terms]) AND "17"[All Fields]) AND "acetyloxy"[All Fields]) AND "6 methyl"[All Fields]) AND "6alpha"[All Fields])) | 66560 |
| 2 | (((((((((((((((((Neoplasm, Ovarian) OR (Ovarian Neoplasm)) OR (Neoplasms, Ovarian)) OR (Ovary Neoplasms)) OR (Neoplasm, Ovary)) OR (Neoplasms, Ovary)) OR (Neoplasms, Ovary)) OR (Ovary Cancer)) OR (Cancer, Ovary)) OR (Cancers, Ovary)) OR (Ovary Cancers)) OR (Cancer of Ovary)) OR (Cancer of the Ovary)) OR (Ovarian Cancer)) OR (Cancer, Ovarian)) OR (Cancers, Ovarian)) OR (Ovarian Cancers)) | "ovarian neoplasms"[MeSH Terms] OR ("ovarian"[All Fields] AND "neoplasms"[All Fields]) OR "ovarian neoplasms"[All Fields] OR ("neoplasm"[All Fields] AND "ovarian"[All Fields]) OR "neoplasm ovarian"[All Fields] OR ("ovarian neoplasms"[MeSH Terms] OR ("ovarian"[All Fields] AND "neoplasms"[All Fields]) OR "ovarian neoplasms"[All Fields] OR ("ovarian"[All Fields] AND "neoplasm"[All Fields]) OR "ovarian neoplasm"[All Fields]) OR ("ovarian neoplasms"[MeSH Terms] OR ("ovarian"[All Fields] AND "neoplasms"[All Fields]) OR "ovarian neoplasms"[All Fields] OR ("neoplasms"[All Fields] AND "ovarian"[All Fields]) OR "neoplasms ovarian"[All Fields]) OR ("ovarian neoplasms"[MeSH Terms] OR ("ovarian"[All Fields] AND "neoplasms"[All Fields]) OR "ovarian neoplasms"[All Fields] OR ("ovary"[All Fields] AND "neoplasms"[All Fields]) OR "ovary neoplasms"[All Fields]) OR ("ovarian neoplasms"[MeSH Terms] OR ("ovarian"[All Fields] AND "neoplasms"[All Fields]) OR "ovarian neoplasms"[All Fields] OR ("neoplasm"[All Fields] AND "ovary"[All Fields]) OR "neoplasm ovary"[All Fields]) OR ("ovarian neoplasms"[MeSH Terms] OR ("ovarian"[All Fields] AND "neoplasms"[All Fields]) OR "ovarian neoplasms"[All Fields] OR ("neoplasms"[All Fields] AND "ovary"[All Fields]) OR "neoplasms ovary"[All Fields]) OR ("ovarian neoplasms"[MeSH Terms] OR ("ovarian"[All Fields] AND "neoplasms"[All Fields]) OR "ovarian neoplasms"[All Fields] OR ("neoplasms"[All Fields] AND "ovary"[All Fields]) OR "neoplasms ovary"[All Fields]) OR ("ovarian neoplasms"[MeSH Terms] OR ("ovarian"[All Fields] AND "neoplasms"[All Fields]) OR "ovarian neoplasms"[All Fields] OR ("ovary"[All Fields] AND "cancer"[All Fields]) OR "ovary cancer"[All Fields]) OR ("ovarian neoplasms"[MeSH Terms] OR ("ovarian"[All Fields] AND "neoplasms"[All Fields]) OR "ovarian neoplasms"[All Fields] OR ("cancer"[All Fields] AND "ovary"[All Fields]) OR "cancer ovary"[All Fields]) OR ("ovarian neoplasms"[MeSH Terms] OR ("ovarian"[All Fields] AND "neoplasms"[All Fields]) OR "ovarian neoplasms"[All Fields] OR ("cancers"[All Fields] AND "ovary"[All Fields]) OR "cancers ovary"[All Fields]) OR ("ovarian neoplasms"[MeSH Terms] OR ("ovarian"[All Fields] AND "neoplasms"[All Fields]) OR "ovarian neoplasms"[All Fields] OR ("ovary"[All Fields] AND "cancers"[All Fields]) OR "ovary cancers"[All Fields]) OR ("ovarian neoplasms"[MeSH Terms] OR ("ovarian"[All Fields] AND "neoplasms"[All Fields]) OR "ovarian neoplasms"[All Fields] OR ("cancer"[All Fields] AND "ovary"[All Fields]) OR "cancer of ovary"[All Fields]) OR ("ovarian neoplasms"[MeSH Terms] OR ("ovarian"[All Fields] AND "neoplasms"[All Fields]) OR "ovarian neoplasms"[All Fields] OR ("cancer"[All Fields] AND "ovary"[All Fields]) OR "cancer of the ovary"[All Fields]) OR ("ovarian neoplasms"[MeSH Terms] OR ("ovarian"[All Fields] AND "neoplasms"[All Fields]) OR "ovarian neoplasms"[All Fields] OR ("ovarian"[All Fields] AND "cancer"[All Fields]) OR "ovarian cancer"[All Fields]) OR ("ovarian neoplasms"[MeSH Terms] OR ("ovarian"[All Fields] AND "neoplasms"[All Fields]) OR "ovarian neoplasms"[All Fields] OR ("cancer"[All Fields] AND "ovarian"[All Fields]) OR "cancer ovarian"[All Fields]) OR ("ovarian neoplasms"[MeSH Terms] OR ("ovarian"[All Fields] AND "neoplasms"[All Fields]) OR "ovarian neoplasms"[All Fields] OR ("cancers"[All Fields] AND "ovarian"[All Fields]) OR "cancers ovarian"[All Fields]) OR ("ovarian neoplasms"[MeSH Terms] OR ("ovarian"[All Fields] AND "neoplasms"[All Fields]) OR "ovarian neoplasms"[All Fields] OR ("ovarian"[All Fields] AND "cancers"[All Fields]) OR "ovarian cancers"[All Fields]) | 162843 |
| 3 | #1 AND #2 | #1 AND #2 | 2029 |

**Web of Science**

| Search number |  | Search Details | Results |
| --- | --- | --- | --- |
| 1 | TS=((((((((((((((((((((((((((((((((((((((Oral Contraceptive) OR (Contraceptive, Oral)) OR (Oral Contraceptives)) OR (Oral Contraceptives, Low-Dose)) OR (Contraceptives, Low-Dose Oral)) OR (Low-Dose Oral Contraceptives)) OR (Low-Dose Oral Contraceptive)) OR (Oral Contraceptives, Low Dose)) OR (Contraceptive, Low-Dose Oral)) OR (Low Dose Oral Contraceptive)) OR (Oral Contraceptive, Low-Dose)) OR (Oral Contraceptives, Phasic)) OR (Contraceptives, Phasic Oral)) OR (Phasic Oral Contraceptives)) OR (Oral Contraceptives, Hormonal)) OR (Contraceptives, Hormonal Oral)) OR (Hormonal Oral Contraceptives)) OR (Contraceptive Agents, Oral, Hormonal)) OR (Hormonal Oral Contraceptive Agents)) OR (Oral Contraceptive Agents, Hormonal)) OR (Hormonal Oral Contraceptive)) OR (Contraceptive, Hormonal Oral)) OR (Oral Contraceptive, Hormonal)) OR (Hormonal Oral Contraceptive Agent)) OR (Contraceptive Agents, Estrogen)) OR (Estrogen Contraceptive Agents)) OR (Combined Oral Contraceptive)) OR (Contraceptive, Combined Oral)) OR (Oral Contraceptive, Combined)) OR (Oral Contraceptives, Combined)) OR (Combined Oral Contraceptives)) OR (Contraceptives, Combined Oral)) OR (Contraceptive Agents, Female, Combined))))))) | TS=(((((((((((((((((((((((((((((((((((((Oral Contraceptive) OR (Oral, Contraceptive) OR (Contraceptive, Oral) OR (Oral Contraceptives) OR (Oral, Contraceptives) OR (Oral Contraceptives, Low-Dose) OR (Contraceptives, Low-Dose Oral) OR (Low-Dose Oral Contraceptives) OR (Low-Dose, Oral, Contraceptives) OR (Low-Dose Oral Contraceptive) OR (Low-Dose, Oral, Contraceptive) OR (Oral Contraceptives, Low Dose) OR (Contraceptive, Low-Dose Oral) OR (Low Dose Oral Contraceptive) OR (Low, Dose, Oral, Contraceptive) OR (Oral Contraceptive, Low-Dose) OR (Oral Contraceptives, Phasic) OR (Contraceptives, Phasic Oral) OR (Phasic Oral Contraceptives) OR (Phasic, Oral, Contraceptives) OR (Oral Contraceptives, Hormonal) OR (Contraceptives, Hormonal Oral) OR (Hormonal Oral Contraceptives) OR (Hormonal, Oral, Contraceptives) OR (Contraceptive Agents, Oral, Hormonal) OR (Hormonal Oral Contraceptive Agents) OR (Hormonal, Oral, Contraceptive, Agents) OR (Oral Contraceptive Agents, Hormonal) OR (Hormonal Oral Contraceptive) OR (Hormonal, Oral, Contraceptive) OR (Contraceptive, Hormonal Oral) OR (Oral Contraceptive, Hormonal) OR (Hormonal Oral Contraceptive Agent) OR (Hormonal, Oral, Contraceptive, Agent) OR (Contraceptive Agents, Estrogen) OR (Estrogen Contraceptive Agents) OR (Estrogen, Contraceptive, Agents) OR (Combined Oral Contraceptive) OR (Combined, Oral, Contraceptive) OR (Contraceptive, Combined Oral) OR (Oral Contraceptive, Combined) OR (Oral Contraceptives, Combined) OR (Combined Oral Contraceptives) OR (Combined, Oral, Contraceptives) OR (Contraceptives, Combined Oral) OR (Contraceptive Agents, Female, Combined))))))) | 57078 |
| 2 | TS=(Neoplasm, Ovarian OR Ovarian Neoplasm OR Neoplasms, Ovarian OR Ovary Neoplasms OR Neoplasm, Ovary OR Neoplasms, Ovary OR Neoplasms, Ovary OR Ovary Cancer OR Cancer, Ovary OR Cancers, Ovary OR Ovary Cancers OR Cancer of Ovary OR Cancer of the Ovary OR Ovarian Cancer OR Cancer, Ovarian OR Cancers, Ovarian OR Ovarian Cancers) | TS=(Neoplasm, Ovarian OR Ovarian Neoplasm OR Ovarian, Neoplasm OR Neoplasms, Ovarian OR Ovary Neoplasms OR Ovary, Neoplasms OR Neoplasm, Ovary OR Neoplasms, Ovary OR Ovary Cancer OR Ovary, Cancer OR Cancer, Ovary OR Cancers, Ovary OR Ovary Cancers OR Ovary, Cancers OR Cancer of Ovary OR Cancer, of, Ovary OR Cancer of the Ovary OR Cancer, of, the, Ovary OR Ovarian Cancer OR Ovarian, Cancer OR Cancer, Ovarian OR Cancers, Ovarian OR Ovarian Cancers OR Ovarian, Cancers) | 257743 |
| 3 | #1 AND #2 | #1 AND #2 | 2780 |

**Ovid**

| Search number | Query | Search Details | Results |
| --- | --- | --- | --- |
| 1 | (Oral Contraceptive OR Contraceptive, Oral OR Oral Contraceptives OR Oral Contraceptives, Low-Dose OR Contraceptives, Low-Dose Oral OR Low-Dose Oral Contraceptives OR Low-Dose Oral Contraceptive OR Oral Contraceptives, Low Dose OR Contraceptive, Low-Dose Oral OR Low Dose Oral Contraceptive OR Oral Contraceptive, Low-Dose OR Oral Contraceptives, Phasic OR Contraceptives, Phasic Oral OR Phasic Oral Contraceptives) OR (Oral Contraceptives, Hormonal OR Contraceptives, Hormonal Oral OR Hormonal Oral Contraceptives OR Contraceptive Agents, Oral, Hormonal OR Hormonal Oral Contraceptive Agents OR Oral Contraceptive Agents, Hormonal OR Hormonal Oral Contraceptive OR Contraceptive, Hormonal Oral OR Oral Contraceptive, Hormonal OR Hormonal Oral Contraceptive Agent OR Contraceptive Agents, Estrogen OR Estrogen Contraceptive Agents) OR (Combined Oral Contraceptive OR Contraceptive, Combined Oral OR Oral Contraceptive, Combined OR Oral Contraceptives, Combined OR Combined Oral Contraceptives OR Contraceptives, Combined Oral OR Contraceptive Agents, Female, Combined) | (Oral Contraceptive OR Contraceptive, Oral OR Oral Contraceptives OR Oral Contraceptives, Low-Dose OR Contraceptives, Low-Dose Oral OR Low-Dose Oral Contraceptives OR Low-Dose Oral Contraceptive OR Oral Contraceptives, Low Dose OR Contraceptive, Low-Dose Oral OR Low Dose Oral Contraceptive OR Oral Contraceptive, Low-Dose OR Oral Contraceptives, Phasic OR Contraceptives, Phasic Oral OR Phasic Oral Contraceptives OR Oral Contraceptives, Hormonal OR Contraceptives, Hormonal Oral OR Hormonal Oral Contraceptives OR Contraceptive Agents, Oral, Hormonal OR Hormonal Oral Contraceptive Agents OR Oral Contraceptive Agents, Hormonal OR Hormonal Oral Contraceptive OR Contraceptive, Hormonal Oral OR Oral Contraceptive, Hormonal OR Hormonal Oral Contraceptive Agent OR Contraceptive Agents, Estrogen OR Estrogen Contraceptive Agents OR Combined Oral Contraceptive OR Contraceptive, Combined Oral OR Oral Contraceptive, Combined OR Oral Contraceptives, Combined OR Combined Oral Contraceptives OR Contraceptives, Combined Oral OR Contraceptive Agents, Female, Combined).m_titl OR (Oral Contraceptive OR Contraceptive, Oral OR Oral Contraceptives OR Oral Contraceptives, Low-Dose OR Contraceptives, Low-Dose Oral OR Low-Dose Oral Contraceptives OR Low-Dose Oral Contraceptive OR Oral Contraceptives, Low Dose OR Contraceptive, Low-Dose Oral OR Low Dose Oral Contraceptive OR Oral Contraceptive, Low-Dose OR Oral Contraceptives, Phasic OR Contraceptives, Phasic Oral OR Phasic Oral Contraceptives OR Oral Contraceptives, Hormonal OR Contraceptives, Hormonal Oral OR Hormonal Oral Contraceptives OR Contraceptive Agents, Oral, Hormonal OR Hormonal Oral Contraceptive Agents OR Oral Contraceptive Agents, Hormonal OR Hormonal Oral Contraceptive OR Contraceptive, Hormonal Oral OR Oral Contraceptive, Hormonal OR Hormonal Oral Contraceptive Agent OR Contraceptive Agents, Estrogen OR Estrogen Contraceptive Agents OR Combined Oral Contraceptive OR Contraceptive, Combined Oral OR Oral Contraceptive, Combined OR Oral Contraceptives, Combined OR Combined Oral Contraceptives OR Contraceptives, Combined Oral OR Contraceptive Agents, Female, Combined).mp. [mp=title, abstract, heading word, drug trade name, original title, device manufacturer, drug manufacturer, device trade name, keyword heading word, floating subheading word, candidate term word] OR exp oral contraceptives/dt, pc, ae, th [Drug Therapy, Pharmacology, Adverse Effects, Therapy] | 138417 |
| 2 | Neoplasm, Ovarian OR Ovarian Neoplasm OR Neoplasms, Ovarian OR Ovary Neoplasms OR Neoplasm, Ovary OR Neoplasms, Ovary OR Neoplasms, Ovary OR Ovary Cancer OR Cancer, Ovary OR Cancers, Ovary OR Ovary Cancers OR Cancer of Ovary OR Cancer of the Ovary OR Ovarian Cancer OR Cancer, Ovarian OR Cancers, Ovarian OR Ovarian Cancers | (Neoplasm, Ovarian OR Ovarian Neoplasm OR Neoplasms, Ovarian OR Ovary Neoplasms OR Neoplasm, Ovary OR Neoplasms, Ovary OR Ovary Cancer OR Cancer, Ovary OR Cancers, Ovary OR Ovary Cancers OR Cancer of Ovary OR Cancer of the Ovary OR Ovarian Cancer OR Cancer, Ovarian OR Cancers, Ovarian OR Ovarian Cancers).m_titl OR (Neoplasm, Ovarian OR Ovarian Neoplasm OR Neoplasms, Ovarian OR Ovary Neoplasms OR Neoplasm, Ovary OR Neoplasms, Ovary OR Ovary Cancer OR Cancer, Ovary OR Cancers, Ovary OR Ovary Cancers OR Cancer of Ovary OR Cancer of the Ovary OR Ovarian Cancer OR Cancer, Ovarian OR Cancers, Ovarian OR Ovarian Cancers).mp. [mp=title, abstract, heading word, drug trade name, original title, device manufacturer, drug manufacturer, device trade name, keyword heading word, floating subheading word, candidate term word] OR exp ovarian neoplasms/di, et, th, pc, pa [Diagnosis, Etiology, Therapy, Prevention, Pathology] | 9859 |
| 3 | #1 AND #2 | #1 AND #2 | 8751 |

**Cochrane**

| Search number | Query | Search Details | Results |
| --- | --- | --- | --- |
| 1 | (Oral Contraceptive OR Contraceptive, Oral OR Oral Contraceptives OR Oral Contraceptives, Low-Dose OR Contraceptives, Low-Dose Oral OR Low-Dose Oral Contraceptives OR Low-Dose Oral Contraceptive OR Oral Contraceptives, Low Dose OR Contraceptive, Low-Dose Oral OR Low Dose Oral Contraceptive OR Oral Contraceptive, Low-Dose OR Oral Contraceptives, Phasic OR Contraceptives, Phasic Oral OR Phasic Oral Contraceptives) OR (Oral Contraceptives, Hormonal OR Contraceptives, Hormonal Oral OR Hormonal Oral Contraceptives OR Contraceptive Agents, Oral, Hormonal OR Hormonal Oral Contraceptive Agents OR Oral Contraceptive Agents, Hormonal OR Hormonal Oral Contraceptive OR Contraceptive, Hormonal Oral OR Oral Contraceptive, Hormonal OR Hormonal Oral Contraceptive Agent OR Contraceptive Agents, Estrogen OR Estrogen Contraceptive Agents) OR (Combined Oral Contraceptive OR Contraceptive, Combined Oral OR Oral Contraceptive, Combined OR Oral Contraceptives, Combined OR Combined Oral Contraceptives OR Contraceptives, Combined Oral OR Contraceptive Agents, Female, Combined) | (Oral Contraceptive):ti,ab,kw OR (Oral, Contraceptive):ti,ab,kw OR (Oral Contraceptives):ti,ab,kw OR (Oral, Contraceptives):ti,ab,kw OR (Oral Contraceptives, Low-Dose):ti,ab,kw OR (Oral Contraceptives Low-Dose):ti,ab,kw OR (Contraceptives, Low-Dose Oral):ti,ab,kw OR (Contraceptives Low-Dose Oral):ti,ab,kw OR  (Low-Dose Oral Contraceptives):ti,ab,kw OR (Low-Dose Oral, Contraceptives):ti,ab,kw OR (Low-Dose Oral Contraceptive):ti,ab,kw OR (Low-Dose Oral, Contraceptive):ti,ab,kw OR (Oral Contraceptives, Low Dose):ti,ab,kw OR (Oral Contraceptives Low Dose):ti,ab,kw OR (Contraceptive, Low-Dose Oral):ti,ab,kw OR (Contraceptive Low-Dose Oral):ti,ab,kw OR (Low Dose Oral Contraceptive):ti,ab,kw OR (Low Dose Oral, Contraceptive):ti,ab,kw OR (Oral Contraceptive, Low-Dose):ti,ab,kw OR (Oral Contraceptive Low-Dose):ti,ab,kw OR (Oral Contraceptives, Phasic):ti,ab,kw OR (Oral Contraceptives Phasic):ti,ab,kw OR (Contraceptives, Phasic Oral):ti,ab,kw OR (Contraceptives Phasic Oral):ti,ab,kw OR (Phasic Oral Contraceptives):ti,ab,kw OR (Phasic Oral, Contraceptives):ti,ab,kw OR (Oral Contraceptives, Hormonal):ti,ab,kw OR (Oral Contraceptives Hormonal):ti,ab,kw OR (Contraceptives, Hormonal Oral):ti,ab,kw OR (Contraceptives Hormonal Oral):ti,ab,kw OR  (Hormonal Oral Contraceptives):ti,ab,kw OR (Hormonal Oral, Contraceptives):ti,ab,kw OR (Contraceptive Agents, Oral, Hormonal):ti,ab,kw OR (Contraceptive Agents Oral Hormonal):ti,ab,kw OR (Hormonal Oral Contraceptive Agents):ti,ab,kw OR (Hormonal Oral, Contraceptive Agents):ti,ab,kw OR (Oral Contraceptive Agents, Hormonal):ti,ab,kw OR (Oral Contraceptive Agents Hormonal):ti,ab,kw OR (Hormonal Oral Contraceptive):ti,ab,kw OR (Hormonal Oral, Contraceptive):ti,ab,kw OR (Contraceptive, Hormonal Oral):ti,ab,kw OR (Contraceptive Hormonal Oral):ti,ab,kw OR (Oral Contraceptive, Hormonal):ti,ab,kw OR (Oral Contraceptive Hormonal):ti,ab,kw OR (Hormonal Oral Contraceptive Agent):ti,ab,kw OR (Hormonal Oral, Contraceptive Agent):ti,ab,kw OR (Contraceptive Agents, Estrogen):ti,ab,kw OR (Contraceptive Agents Estrogen):ti,ab,kw OR (Estrogen Contraceptive Agents):ti,ab,kw OR (Estrogen, Contraceptive Agents):ti,ab,kw OR (Combined Oral Contraceptive):ti,ab,kw OR (Combined Oral, Contraceptive):ti,ab,kw OR (Contraceptive, Combined Oral):ti,ab,kw OR (Contraceptive Combined Oral):ti,ab,kw OR  (Oral Contraceptive, Combined):ti,ab,kw OR (Oral Contraceptive Combined):ti,ab,kw OR (Oral Contraceptives, Combined):ti,ab,kw OR (Oral Contraceptives Combined):ti,ab,kw OR (Combined Oral Contraceptives):ti,ab,kw OR (Combined Oral, Contraceptives):ti,ab,kw OR (Contraceptives, Combined Oral):ti,ab,kw OR (Contraceptives Combined Oral):ti,ab,kw OR  (Contraceptive Agents, Female, Combined):ti,ab,kw OR(Contraceptive Agents Female Combined):ti,ab,kw | 9501 |
| 2 | Neoplasm, Ovarian OR Ovarian Neoplasm OR Neoplasms, Ovarian OR Ovary Neoplasms OR Neoplasm, Ovary OR Neoplasms, Ovary OR Neoplasms, Ovary OR Ovary Cancer OR Cancer, Ovary OR Cancers, Ovary OR Ovary Cancers OR Cancer of Ovary OR Cancer of the Ovary OR Ovarian Cancer OR Cancer, Ovarian OR Cancers, Ovarian OR Ovarian Cancers | (Neoplasm, Ovarian):ti,ab,kw OR (Neoplasm Ovarian):ti,ab,kw OR (Ovarian Neoplasm):ti,ab,kw OR (Ovarian, Neoplasm):ti,ab,kw OR (Neoplasms, Ovarian):ti,ab,kw OR (Neoplasms Ovarian):ti,ab,kw OR (Ovary Neoplasms):ti,ab,kw OR (Ovary, Neoplasms):ti,ab,kw OR (Neoplasm, Ovary):ti,ab,kw OR (Neoplasm Ovary):ti,ab,kw OR  (Neoplasms, Ovary):ti,ab,kw OR (Neoplasms Ovary):ti,ab,kw OR (Ovary Cancer):ti,ab,kw OR (Ovary, Cancer):ti,ab,kw OR (Cancer, Ovary):ti,ab,kw OR (Cancer Ovary):ti,ab,kw OR (Cancers, Ovary):ti,ab,kw OR (Cancers Ovary):ti,ab,kw OR (Ovary Cancers):ti,ab,kw OR (Ovary, Cancers):ti,ab,kw OR (Cancer of Ovary):ti,ab,kw OR (Cancer of, Ovary):ti,ab,kw OR  (Cancer of the Ovary):ti,ab,kw OR (Cancer of the, Ovary):ti,ab,kw OR (Ovarian Cancer):ti,ab,kw OR (Ovarian, Cancer):ti,ab,kw OR (Cancer, Ovarian):ti,ab,kw OR (Cancer Ovarian):ti,ab,kw OR (Cancers, Ovarian):ti,ab,kw OR (Cancers Ovarian):ti,ab,kw OR (Ovarian Cancers):ti,ab,kw OR (Ovarian, Cancers):ti,ab,kw | 10324 |
| 3 | #1 AND #2 | #1 AND #2 | 82 |
