# Supplementary material for: Oral contraceptives exposure may reduce the risk of ovarian cancer: a meta-analysis based on cohort studies
Source: Front Pharmacol. 2026 Apr 1;17:1732719. doi: 10.3389/fphar.2026.1732719 (PMC13079376; doi:10.3389/fphar.2026.1732719)
Supplement: Supplementary file 4 [file Table2.doc]

**Supplementary Table 2. Quality assessment of the cohort studies included.**

| Author, year | **Selection (Out of 4)** | | | | **Comparability**  **(Out of 2)** | **Outcomes (Out of 3)** | | | **Total**  **(Out of 9)** |
| --- | --- | --- | --- | --- | --- | --- | --- | --- | --- |
|  | Representativeness of exposed cohort | Selection of non exposed cohort | Ascertainment  of exposure | Outcome not present at the start of the study |  | Assessment of outcomes | Length of follow-up | Adequacy of follow up of cohorts |  |
| Karlsson T, 2021 | 1 | 1 | 1 | 1 | 1 | 1 | 1 | 1 | 8 |
| Bethea T N, 2017 | 1 | 1 | 1 | 1 | 2 | 1 | 1 | 0 | 8 |
| Hippisley-Cox J, 2015 | 1 | 1 | 1 | 1 | 1 | 1 | 1 | 0 | 7 |
| Braem M G, 2010 | 1 | 1 | 1 | 1 | 2 | 1 | 1 | 1 | 9 |
| Gay G M, 2015 | 1 | 1 | 1 | 1 | 1 | 1 | 1 | 1 | 8 |
| Sarink D, 2020 | 1 | 1 | 1 | 1 | 1 | 1 | 1 | 1 | 8 |
| Laaksonen M A, 2019 | 1 | 1 | 1 | 1 | 1 | 1 | 1 | 0 | 7 |
| McGuire V, 2016 | 1 | 1 | 1 | 1 | 1 | 1 | 1 | 0 | 7 |
| Shafrir A L, 2017 | 1 | 1 | 1 | 1 | 2 | 1 | 1 | 1 | 9 |
| Fortner R T, 2015 | 1 | 1 | 1 | 1 | 2 | 1 | 1 | 1 | 9 |
| Huang Z, 2015 | 1 | 1 | 1 | 1 | 2 | 1 | 1 | 1 | 9 |

The cohort studies were assessed by the Newcastle-Ottawa Quality Assessment Scale (NOS) checklist.
